# Elucidating the Boundary of Intercalation vs Sequestration in Supramolecular Polymers by Retrosynthetic Design Toward the Construction of Complex Supramolecular Systems

**DOI:** 10.1002/anie.202501693

**Published:** 2025-03-10

**Authors:** Nils Bäumer, Soichiro Ogi, Shigehiro Yamaguchi

**Affiliations:** ^1^ Institute of Transformative Bio‐Molecules (WPI‐ITbM) Nagoya University Furo, Chikusa Nagoya 464–8601 Japan; ^2^ Integrated Research Consortium on Chemical Science (IRCCS) Nagoya University Furo, Chikusa Nagoya 464–8602 Japan; ^3^ Department of Chemistry Graduate School of Science Nagoya University Furo Chikusa Nagoya 464–8602 Japan

**Keywords:** Intercalation, Retrosynthetic analysis, Sequestration, Supramolecular polymerization, Systems chemistry

## Abstract

Controlled social self‐sorting by intercalation can offer distinct properties at the supramolecular level that go beyond the sum of its parts. Likewise, controlling narcissistic self‐sorting by sequestration can induce unique system properties. In contrast, the interface between the two cases has hitherto remained underexplored, and clear design rules remain elusive. Herein it is demonstrated that by fine‐tuning the molecular similarity of supramolecular synthons, intricate control over concerted supramolecular equilibria can be achieved. By reducing the molecular similarity, a former intercalator can be tuned to become a strong or weak sequestrator. Understanding these roles in binary mixtures allows to rationalize more complex tertiary systems. Consequently, the influence of an uncommon dual sequestration mechanism is revealed. Further, an unprecedented hybrid mechanism between supramolecular intercalation and sequestration can be demonstrated. We are hopeful that the results presented herein will contribute to the development and understanding of concerted processes in complex supramolecular systems.

## Graphical abstract

By retrosynthetically analyzing the molecular structure of an intercalator, clear design principles toward supramolecular sequestration can be obtained. Investigating a model supramolecular polymer in the presence of these additives offers a general approach toward dilution‐induced polymerization and complex hybrid systems of dual sequestration and hybrid mechanisms of sequestration and intercalation.

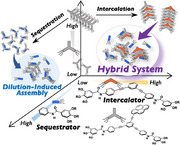

## Introduction

In recent years much effort in supramolecular polymer research has been dedicated to moving from simple one‐component to more complex mixtures.^[^ ^1^, ^2^ ^]^ Inspired by the highly adaptive assemblies found in complex natural environments, these systems hold great promise as the next generation of adaptive soft matter. In this context, social self‐sorting has received particular attention as it can enable access to functional properties, that are inaccessible for each building block in isolation.^[^ ^3^, ^4^ ^]^ These properties include, but are not limited to unique photoluminescence phenomena,^[^ ^3^ ^]^ enhanced supramolecular stability^[^ ^5^ ^]^ as well as hybrid topologies.^[^ ^6^, ^7^ ^]^ In addition to offering potentially attractive properties, social self‐sorting has another intriguing benefit, namely its relatively simple actualization. By combining different supramolecular synthons with a high degree of similarity, i.e., a nearly identical molecular structure, statistical co‐polymerization by intercalation can be readily achieved.^[^ ^8^ ^]^ Further in‐depth control over the nanoscopic order within a supramolecular polymer can be achieved by utilizing specific intermolecular binding patterns,^[^ ^9^, ^10^, ^11^ ^]^ that enforce an alternating arrangement. Additionally, by altering the social intermolecular binding strengths a supramolecular “blocky” or block co‐polymerization can be realized.^[^ ^8^ ^]^ In this context assembly protocols,^[^ ^5^, ^12^, ^13^, ^14^ ^]^ rather than molecular design have also proven to be highly effective, with seed‐initiated polymerization being one of the most common approaches to implement specific sequential growth regimes under kinetic control.^[^ ^15^ ^]^

The other extreme of the spectrum, which is fully narcissistic self‐sorting, can likewise offer appealing research avenues. For example, natural systems represent a highly diverse combination of macromolecular entities in addition to their smaller molecular building blocks. Nevertheless, functional natural assemblies, such as protein complexes^[^ ^16^ ^]^ require precise assembly pathways, that are free of social interactions with other potential binding partners to maintain their intricate functionality.^[^ ^17^ ^]^ If researchers wish to mimic such complex systems, each chosen building block must show an equally robust assembly behavior.^[^ ^18^ ^]^ From a molecular design aspect, this can also be realized reliably, as a mismatch in the supramolecular binding pattern can enforce narcissistic assembly pathways.^[^ ^19^ ^]^ On the other hand, even if the primary elongation of two supramolecular synthons is incompatible secondary phenomena, such as surface‐catalyzed secondary nucleation^[^ ^20^ ^]^ or crowding effects^[^ ^21^ ^]^ may arise in multicomponent mixtures, making their practical implications at times unpredictable.

The interface in between the two extreme cases, that is compounds that can form intermolecular contacts, but are unable to efficiently elongate into co‐assembled structures, has remained underexplored so far.^[^ ^22^ ^]^ In these systems, the individual building blocks can interact with each other to form discrete dimer/trimer structures or short oligomers, but unrestricted elongation is suppressed. Such behavior has been demonstrated using geometrically incompatible building blocks,^[^ ^23^ ^]^ by fine‐tuning steric demand,^[^ ^24^ ^]^ or by blocking intermolecular binding sites.^[^ ^25^ ^]^ Similarly, adaptive behavior in these systems could be achieved by modulating steric profiles using covalent^[^ ^26^ ^]^ and light induced transformations in situ.^[^ ^27^ ^]^ However, the boundaries of molecular similarity that dictate whether an additive can act as an intercalator or sequestrator remain elusive. As multi‐component systems are gaining wide scale interest these clear structure‐property relationships are urgently required to accelerate their development.^[^ ^18^ ^]^

**Figure 1:**
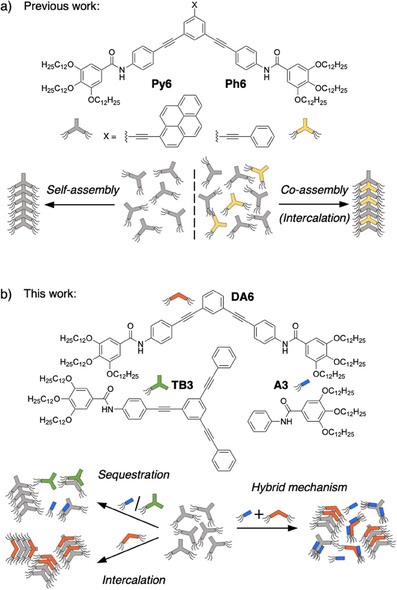
a) Molecular structure and schematic representation of the social self‐sorting behavior of **Py6** and **Ph6**. b) Molecular structures of **DA6**, **TB3**, and **A3** used in this study and a schematic representation of their narcissistic and social self‐sorting behavior.

In this context, we have recently reported on a supramolecular modulator strategy, where a phenylacetylene appended benzene diamide (**Ph6**, Figure 1a) can undergo fully social self‐sorting with a pyrene appended analog (**Py6**) due to its ability to randomly intercalate into the polymer of **Py6** at any ratio of the two comonomers.^[^ ^28^ ^]^ This concentration‐independent intercalation behavior was found to be driven by the compatibility in intermolecular binding patterns between the two synthons. In this study, by using a retrosynthetic design strategy we have designed a series of additives that can act either as a concentration‐dependent intercalator/sequestrator or as sequestrators with differing fidelity. More specifically, by removing the pyrene group we obtained an additive that shares the central bent oligo(phenylene‐ethynylene)‐diamide structure with a total of six solubilizing chains with **Py6** and can act as a concentration‐dependent intercalator (**DA6**, Figure 1b). Conversely, by removing one amide moiety the structure retains the central trisubstituted benzene structure with one amide group and three solubilizing chains (**TB3**). Further reduction of the molecular similarity removes the conjugated aromatic scaffold leaving only a simple diphenyl amide with the solubilizing groups (**A3**), leading to two different sequestrators with differing binding strengths to **Py6**. Our results thus highlight clear design principles, which govern concerted supramolecular equilibria and their corresponding assembly or disassembly pathways. Consideration of the underlying mass balance equations allows a general approach toward dilution‐induced self‐assembly. Further, the design of complex multicomponent systems, where an intercalator and a sequestrator compete for interactions with a model supramolecular polymer, as well as dual sequestration mechanisms could be demonstrated.

## Results and Discussion

### Synthesis

The compounds **A3**, **TB3**, and **DA6** have been synthesized following standard protocols based on sequential Sonogashira coupling and deprotection reactions, identical to those previously used for the synthesis of **Py6**. Synthetic details and the characterization of all new compounds are included in the Supporting Information (SI) of this manuscript.

### Impact of Molecular Design on One‐Component Self‐Assembly

**Figure 2:**
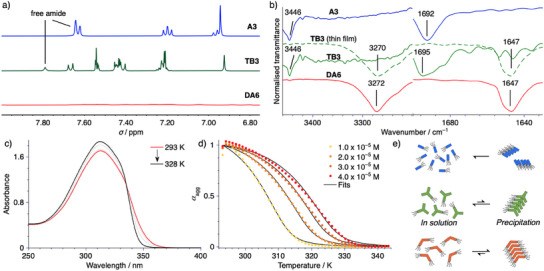
a) Partial ^1^H NMR spectra of **A3** (blue, *c* = 1.0 × 10^−3^ m), **TB3** (green, *c* = 5.0 × 10^−4^ m), and **DA6** (red, *c* = 5.0 × 10^−4^ m) at *T* = 293 K in cyclohexane‐*d*_12_; the intensity was adjusted to the solvent peak of cyclohexane‐*d*_12_ for comparison. b) FT–IR spectra of **A3** (blue), **TB3** (green), and **DA6** (red) at *c* = 5.0 × 10^−4^ m and *T* = 293 K in hexane as well as the thin film of the precipitate of **TB3** (green dashed line) obtained from drop‐casting *V* = 100 µL of a suspension of **TB3** formed at *c* = 1.0 × 10^−3^ m in hexane. c) Temperature‐dependent UV absorption spectra of **DA6** at *c* = 2.0 × 10^−5^ m in hexane between *T* = 293 and 328 K using a heating rate of 1 K min^−1^. d) Changes in *α*_agg_ plotted against the temperature for multiple concentrations with fits to the nucleation‐elongation model using a global fitting approach. e) Schematic representation of the assembly behavior of **A3** (blue), **TB3** (green), and **DA6** (red) in hexane at *T* = 293 K and *c* = 5.0 × 10^−4^ m corresponding to the conditions used for FT–IR.

Before investigating more complex mixtures we first studied the additives in isolation using a combined approach of ^1^H nuclear magnetic resonance (NMR), Fourier transform infrared (FT–IR), and time‐/solvent‐/temperature‐dependent UV absorption spectroscopy (Figure 2; Figure S1). Based on the molecular design it can be expected that the binding constant of **DA6** will be higher than those of **A3** and **TB3**. This suggests that **A3** and **TB3** may exist in a molecularly dissolved state in apolar media, even at higher concentrations. To probe this, we employed ^1^H NMR in cyclohexane‐*d*_12_ at *c* = 1.0 × 10^−3^ m for **A3** and *c* = 5.0 × 10^−4^ m for **TB3** and **DA6** (Figure 2a). Both **A3** and **TB3** show well‐resolved proton signals even in pure cyclohexane‐*d*_12_, which are comparable to those observed during characterization in CDCl_3_ (see synthetic details). We note that even the signals of the amide protons remain well resolved at 7.64 and 7.79 ppm for **A3** and **TB3** respectively in apolar environments (the amide signal of **A3** overlaps with another aromatic proton), which is highly enthalpically unfavorable.^[^ ^29^ ^]^ Analogous studies with **DA6** reveal a drastically different behavior, as a near complete vanishing of the signals can be appreciated (Figure 2a), indicating the supramolecular polymerization under these experimental conditions. These results are a clear indication of the pronounced intermolecular binding strength of **DA6**.

To probe the different hydrogen bonding abilities in more detail we conducted FT–IR studies in hexane using a concentration of *c* = 5.0 × 10^−4^ m (Figure 2b). The main bands observed for both **A3** and **TB3** are in good agreement with previously reported examples of “free” (i.e., not intermolecularly bonded) C═O and N─H stretching frequencies.^[^ ^30^, ^31^ ^]^ However, during the sample preparation of **TB3** a partial precipitation of the sample could be observed upon prolonged equilibration, resulting in a slightly lower signal‐to‐noise ratio in the FT–IR spectra. Using thin‐film samples prepared from the precipitates reveals FT–IR bands that can be readily attributed to intermolecular amide‐amide hydrogen bonding.^[^ ^32^ ^]^ Time‐dependent UV absorption spectroscopy (Figure S2) showed that the formation of these hierarchical assemblies is accelerated at higher concentrations, indicating that even though the process is kinetically controlled, it does not involve a distinct off‐pathway species. Analyzing thin film samples of **TB3** by UV–vis reveals typical broadening of the absorption maximum (Figure S3).^[^ ^33^ ^]^ Transmission electron microscopy (TEM) could reveal the morphology of these macroscopic structures as hierarchical spherical assemblies of highly entangled 1D fibers (Figure S4). According to the combined analysis by ^1^H NMR and FT–IR, we conclude that the monomers of **A3** have a relatively high solubility in apolar media. In the case of **TB3**, the potential enthalpic gain upon assembly is evidenced by the formation of poorly dissolved macroscopic assemblies in equilibrium with the monomer.

The supramolecular equilibrium of **DA6** under the experimental conditions used for FT–IR is dominated by the assembled state, evidenced by the near identical stretching frequencies to those of the precipitate of **TB3**. This assignment is corroborated by TEM analysis revealing either spherical or fibrous assemblies depending on the concentration in the apolar medium (Figure S5). We sought to investigate the thermodynamic stability of the supramolecular polymer in more detail. To this end, we employed temperature‐dependent UV absorption spectroscopy under dilute conditions (*c* = 1.0–4.0 × 10^−5^ m, Figure 2c; Figure S6). The disassembly process of **DA6** aggregates is characterized by a decrease in the absorption at lower energies and a concomitant increase in the absorption maximum at *λ*_abs_ = 310 nm which coincides with a clear isosbestic point at *λ*_abs_ = 335 nm. These spectral changes match those of structurally related compounds, suggesting an analogous molecular packing stabilized by (weak) aromatic interactions and two‐fold hydrogen bonding.^[^ ^28^, ^34^, ^35^, ^36^ ^]^ Likely, the absence of a clear shift in the absorption maximum is caused by a rotational displacement between neighboring molecules within the supramolecular ensemble to allow for efficient hydrogen bonding, leading to weak inter‐chromophore coupling. To gain insights into the assembly mechanism we extracted the changes in absorbance at *λ*_abs_ = 345 nm and calculated the corresponding aggregation parameter (*α*_agg_, Figure 2d; Figure S6). The nucleation‐elongation (cooperative) model by ten Eikelder et al.^[^ ^37^ ^]^ proved successful in reproducing the experimental data using a global fitting approach, indicating that the system exists under thermodynamic equilibrium throughout the disassembly process (Table S1). To summarize, **DA6** assembles cooperatively in nonpolar media. These assemblies are stabilized by two‐fold hydrogen bonding, with additional enthalpic contributions from (weak) aromatic interactions as well as van der Waals interactions between the peripheral alkoxy chains.

In short, both **A3** and **TB3** exist in a molecularly dissolved state in non‐polar media at low concentrations. While **A3** retains a high solubility at elevated concentrations **TB3** partially forms aggregates under these conditions, which tend to precipitate from the solution (Figure 2e). Owing to the enthalpically highly unfavorable exposure of the amide moiety to the solvent medium we anticipate that both compounds may act as a sequestrator for the assembly of **Py6** in binary mixtures. In contrast, **DA6** readily assembles in hexane even at concentrations as low as *c* = 1.0 × 10^−5^ m (Figure S6). Crucially, the supramolecular packing mode of these assemblies was found to be stabilized by identical intermolecular interactions as those of **Py6**. Consequently, we speculate that **DA6** may show intercalation behavior with **Py6**.

### Self‐Assembly in Two‐Component Systems and Dilution‐Induced Enhanced Polymerization

**Figure 3:**
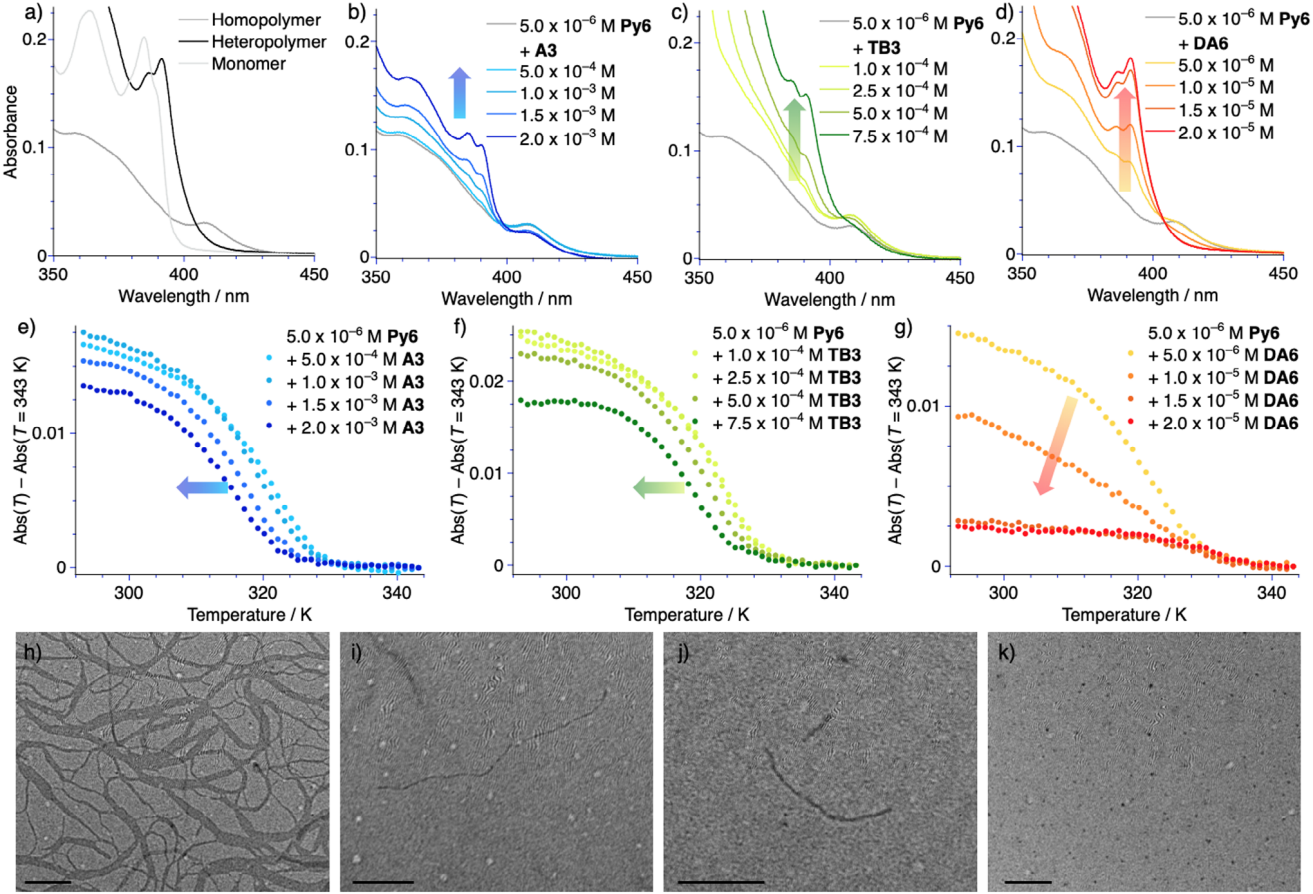
a) UV–vis absorption spectra of **Py6** at *c* = 5.0 × 10^−6^ m in hexane in the homopolymer at *T* = 293 K, molecularly dissolved state at *T* = 343 K, and heteropolymer at *T* = 293 K in the presence of **DA6** at *c* = 2.0 × 10^−5^ m. b–d) UV–vis absorption spectra of **Py6** at *c* = 5.0 × 10^−6^ m in the presence of varying amount of **A3** (b), **TB3** c) and **DA6** d) in hexane at *T* = 293 K. e–g) Relative changes in absorbance at *λ*_abs_ = 415 nm plotted against the temperature between *T* = 293 and 343 K extracted from temperature‐dependent heating studies of the solutions shown in b–d; heating rate: 1 K min^−1^; measurement interval: 1 K. h–k) TEM micrographs of **Py6** at *c* = 5.0 × 10^−6^ m in isolation (h), in the presence of **A3** at *c* = 2.0 × 10^−3^ m i); **TB3** at *c* = 1.0 × 10^−3^ m j) and **DA6** at *c* = 2.0 × 10^−5^ m k) obtained after dropcasting (*V* = 10 µL) from a hexane solution; scale bars correspond to 200 nm.

Following their study in isolation, we probed the influence of the different additives on the self‐assembly of **Py6** in the presence of varying amounts of the different additives. Thus, we investigated samples containing **Py6** at a fixed concentration of *c* = 5.0 × 10^−6^ m in hexane by UV–vis absorption spectroscopy at *T* = 293 K (Figure 3a–d). In brief, the homopolymer of **Py6** is characterized by a broad absorption peak centered at 408 nm, while the heteropolymer has a characteristic absorption peak at 391 nm with a low energy shoulder at 386 nm (Figure 3a). Conversely, the molecularly dissolved state shows an inverse peak shape with a maximum at 385 nm and a high energy shoulder ≈390 nm (Figure 3a). Informed by the self‐assembly studies of the different additives in isolation we increased the amount of additive in a step‐wise fashion (Δ*c* = 5.0 × 10^−4^ m for **A3**, Δ*c* = 2.5 × 10^−4^ m for **TB3** with an additional intermediate additive loading of *c* = 1.0 × 10^−4^ m and Δ*c* = 5.0 × 10^−6^ m for **DA6**). In the case of **A3**, comparatively small changes in the spectrum can be observed even when the additive is present at higher concentrations (Figure 3b). Under these conditions, a minor decrease in the absorption intensity at 408 nm, which is characteristic of the homopolymer of **Py6** can be observed, which coincides with an increase of a double band at 385 and 390 nm. This change in absorbance indicates that the addition of **A3** shifts the supramolecular equilibrium of **Py6** toward the depolymerized state. A similar trend can be observed in binary mixtures of **Py6** and **TB3**, albeit the spectroscopic trends are more pronounced compared to **A3**, even under significantly lower additive loading (Figure 3c). This observation can be readily explained by the higher molecular similarity between **Py6** and **TB3**, compared to **A3**, which leads to a higher binding affinity. Even when **TB3** is present at a high enough concentration to induce its partial supramolecular homo‐polymerization (*c* > 2.5 × 10^−4^ m), no spectroscopic signature of the socially self‐sorted copolymer of **Py6** and **TB3** could be detected. We argue that the bent benzene diamide structure in **Py6** is a prerequisite to induce an efficient co‐assembly process, as the lack of dual hydrogen bonding would result in the exposure of the second amide moiety to the non‐polar solvent medium, which comes at a high enthalpic cost. These considerations lead to the conclusion that a compound equipped with this dual hydrogen bonding structure would be able to engage in social self‐sorting with **Py6** and result in the characteristic absorption changes, associated with its copolymerization process. Accordingly, even at comparatively dilute additive concentrations of **DA6** (*c* = 1.5 × 10^−5^ m) we observed the rise of the distinct absorption band of **Py6** in a copolymer environment with a maximum at 391 nm (Figure 3d), which is consistent with previous observations.^[^ ^28^ ^]^ According to these observations, **DA6** likely acts as an intercalator with **Py6**. These results emphasize the importance of the central benzene diamide core to facilitate access to co‐assembly pathways in the present system and offer a clear structure‐property relationship.

To deepen our understanding of the influence of the different additives we conducted temperature‐dependent UV–vis studies, using identical conditions as those described in the previous section (Figure 3e–g; Figures S7–S9). As none of the additives show significant absorption in the spectral region, where the homopolymer shows a significantly stronger absorbance compared to the molecularly dissolved state and the heteropolymer species (*λ*_abs_ = 415 nm), we can exclusively track the assembly status of **Py6** by monitoring this wavelength. It can be noted that in the case of **A3** as well as **TB3** a nearly analogous change of the secondary plot can be observed (Figure 3e,f). Namely, the elongation temperature is shifted to lower temperatures with increasing concentration of the additive. However, in the case of **A3**, a substantially larger amount of the additive is required to achieve a comparable influence on the elongation temperature as in analogous experiments using **TB3** (Table S2). This experimental result is consistent with previous qualitative assessments suggesting a higher sequestration ability of **TB3** compared to **A3**. In contrast, the influence of **DA6** has to be discussed in two separate concentration regimes. Under relatively low additive concentrations (*c* ≤ 1.0 × 10^−5^ m) the influence of **DA6** can be interpreted to resemble those of **A3** and **TB3**, i.e., that of a sequestrator. The observed threshold concentration aligns well with the transition between the nucleation and elongation regime of the homo‐polymerization of **DA6** in isolation (Figure S10), suggesting that the concerted equilibria may be approximated from their constituents. Above this apparent threshold concentration, the spectral signature of the homo‐polymer is lost and the heteropolymer becomes the dominant species, which is reflected in the near complete depletion of the absorbance at *λ*_abs_ = 415 nm (Figure 3g; Figure S9) even at temperatures below the elongation temperature of 327 K for the homopolymer of **Py6**. This observation indicates that **DA6** can act as either sequestrator or intercalator, depending on the concentration.

To support these assignments, we turned to TEM. In isolation, **Py6** forms highly flexible fibers that are multiple microns in length even under highly dilute conditions (*c* = 5.0 × 10^−6^ m, Figure 3h). In the presence of the sequestrators **A3** and **TB3** at high concentrations this elongation behavior is suppressed and only significantly shorter fragments of fibers could be detected (Figure 3i,j), indicating that **Py6** is partially sequestered in concerted equilibria with **A3** and **TB3**. In binary mixtures with **DA6**, even low additive concentrations are sufficient to completely suppress the fiber formation of **Py6** and instead, only small spherical assemblies could be observed, which resemble those of **DA6** at identical concentrations in isolation (Figure 3k). These observations in the dried state could be qualitatively supported by dynamic light scattering (DLS) in solution, revealing a more drastic decrease in the correlation function in the presence of **DA6** compared to **A3** and **TB3** (Figure S11).

**Figure 4:**
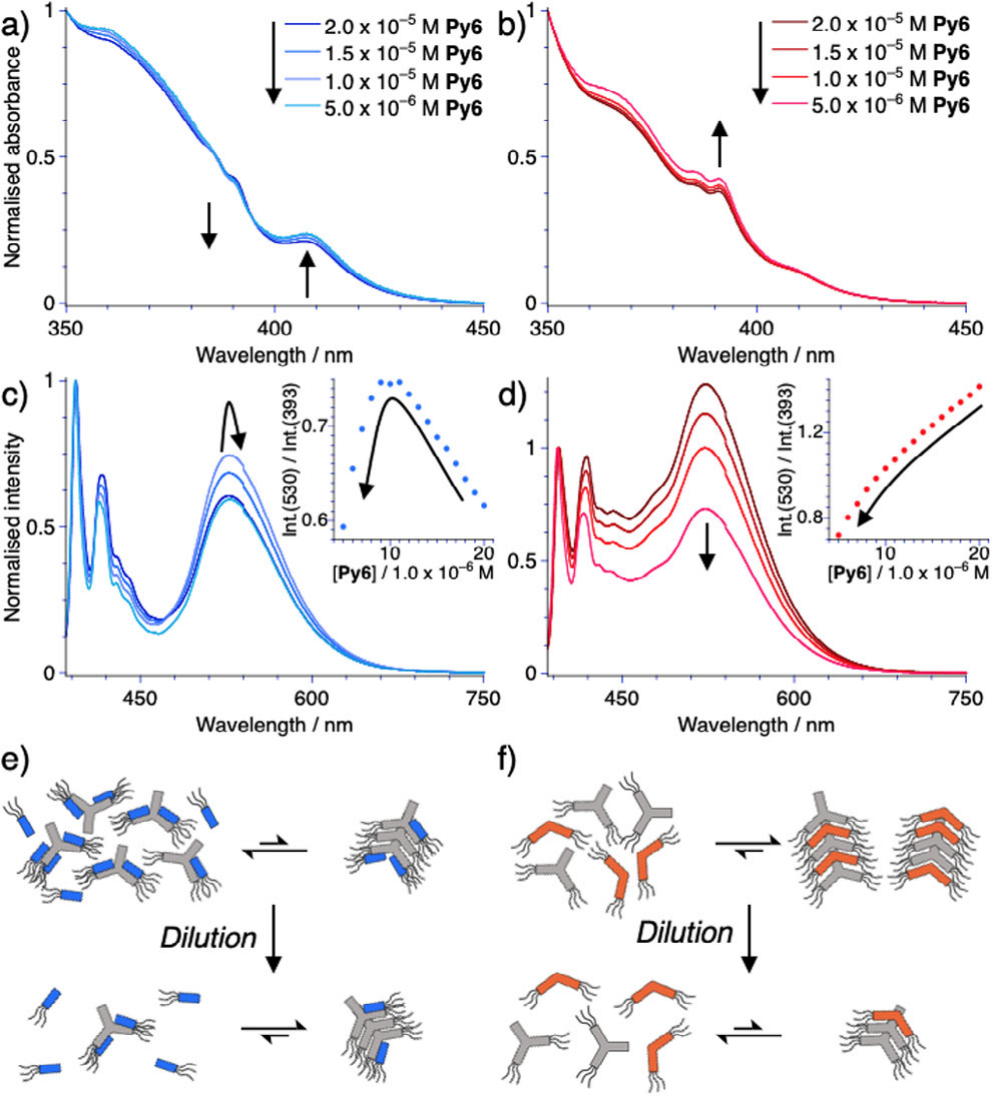
a–d) Normalized concentration‐dependent UV–vis absorption (a,b) and photoluminescence (*λ*_ex_ = 375 nm; c,d) spectra of **Py6** in the presence of 100 eq. of **A3** (a,c), and 1 eq. of **DA6** (b,d) in hexane at *T* = 298 K. Insets show the intensity ratio between 530 and 393 nm. Arrows indicate the concentration‐dependent spectral changes upon dilution. e, f) Schematic representation of dilution‐induced self‐assembly of **Py6** in the presence of **A3** (e) and dilution‐induced disassembly of **Py6** in the presence of **DA6** (f).

Based on these results it appears conceivable that the concerted supramolecular equilibria in binary mixtures of **Py6** and **A3** may behave opposite to those of **Py6** and **DA6** upon dilution, i.e., that mixtures of **Py6** and **A3** may show dilution‐induced supramolecular polymerization. This phenomenon has to the best of our knowledge thus far only been observed for small molecule sequestrators^[^ ^38^, ^39^ ^]^ as well as for systems with a high degree of molecular similarity.^[^ ^25^ ^]^ Further insight into this intriguing phenomenon appears necessary. To this end, we prepared samples of **Py6** (*c* = 2.0 × 10^−5^ m) in the presence of **A3** (*c* = 2.0 × 10^−3^ m) or **DA6** (*c* = 2.0 × 10^−5^ m) and diluted the samples with hexane. By using a combined approach of UV–vis absorption and photoluminescence spectroscopy we followed the supramolecular evolution in detail (Figure 4). As anticipated the spectral changes indicate that diluting the binary mixtures involving the sequestrator **A3** initially leads to a slight shift of the supramolecular equilibrium toward the homopolymer of **Py6**, followed by a reversion of these spectral changes upon further dilution (Figure 4a,c).

Given the overall small spectral changes, the system under investigation may be more accurately described as showing dilution‐induced enhanced polymerization (see Discussion S1). In contrast, the mixture of **Py6** with its intercalator **DA6** shows more traditional dilution‐induced disassembly (Figure 4b,d), which is accelerated under the threshold concentration for the homo‐polymerization of **DA6** (*c* ≤ 1.0 × 10^−5^ m). These observations offer corroborating support for the assignments of the different additives.

### Intercalation vs Sequestration in Three‐Component Systems

**Figure 5:**
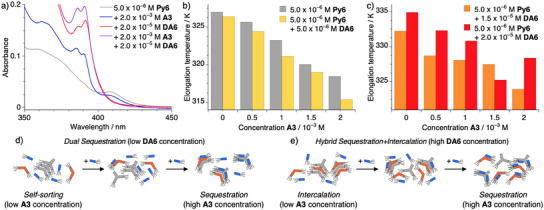
a) UV–vis absorption spectra of **Py6** at *c* = 5.0 × 10^−6^ m in the presence of the varying amount of **A3**, **DA6** and a combination of both in hexane at *T* = 293 K. b, c) Elongation temperature of the homopolymer of **Py6** (determined at *λ*_abs_ = 415 nm, b) and the heteropolymer of **Py6** and **DA6** (determined at *λ*_abs_ = 395 nm, c) using varying concentrations of **DA6** plotted against the concentration of **A3**. d,e) Schematic representation of the dominant concerted supramolecular equilibrium state using low (e) or high f) concentrations of **DA6** upon formal increase in the concentration of **A3**.

Following this analysis in binary mixtures we were intrigued to find out if the influence of the additives can also be carried over to multicomponent mixtures and to unravel if these effects are additive, or counteract each other (Figure 5a–c). Specifically, we have chosen to focus on tertiary mixtures between **Py6**, **A3**, and **DA6** using identical concentration as in the previous experiments, i.e., the concentration of **A3** is increased in steps of 5.0 × 10^−4^ m and that of **DA6** in steps of 5.0 × 10^−5^ m with the concentration of **Py6** remaining fixed at *c* = 5.0 × 10^−6^ m leading to 25 different mixtures overall (Figures S13–S17). We have chosen to focus on **A3** as a sequestrator due to the aforementioned pronounced solubility of the molecularly dissolved state (Figures S2, S4). In order to quantify the influence of the additives the secondary plots have been fitted to extract the *T*_e_ using a wavelength that is either characteristic for the homopolymer of **Py6** (*λ*_abs_ = 415 nm) or the heteropolymer of **Py6** and **DA6** (*λ*_abs_ = 395 nm), depending on the specific concentration of **DA6** (Figure 5b,c; Figures S13–S17). It should be noted that other thermodynamic parameters that the fitting procedure provides should be interpreted with care (Figure S13b), as their determination relies on mass balance equations of single component systems, which is unsuitable in the present study.^[^ ^37^ ^]^ However, making use of the copolymerization model allowed us to estimate the thermodynamic parameters of the co‐assembly process and the obtained fits were able to reproduce the observed experimental behavior (Figure S18,Table S3).^[^ ^40^ ^]^

By comparing all independent experiments, it can be observed that when **DA6** is added at a low concentration of 5.0 × 10^−6^ m the influences of both additives complement each other and they concurrently act as sequestrators in the polymerization of **Py6** in a dual mechanism (Figure 5d). This conclusion is evidenced by the pronounced decrease in the elongation temperature in the presence of both **DA6** and **A3**, compared to the control in binary mixtures of **Py6** and **A3** (Figure 5b). In contrast, an opposite influence was observed, when the concentration of **DA6** exceeds a critical threshold concentration (*c* > 1.0 × 10^−5^ m, Figure 5c). By further increasing the concentration of **DA6** the elongation temperature of the now spectroscopically dominant copolymer of **Py6** and **DA6** increases (Figure 5c). These observations can be readily rationalized by a competitive hybrid mechanism featuring concerted equilibria in which **A3** acts as a sequestrator for the disassembly of **Py6**, while **DA6** can co‐assemble as an intercalator with **Py6** (Figure 5e). It should be noted that using intermediate concentrations of **DA6** leads to more complex behavior, where both the homopolymer of **Py6** as well as its co‐assembled state with **DA6** contribute to the overall absorption profile (Figure S15). This observation is reminiscent of supramolecular packing polymorphism in single component systems and can be attributed to a small energy difference between both assembled states under these conditions.^[^ ^32^, ^41^, ^42^ ^]^

**Figure 6:**
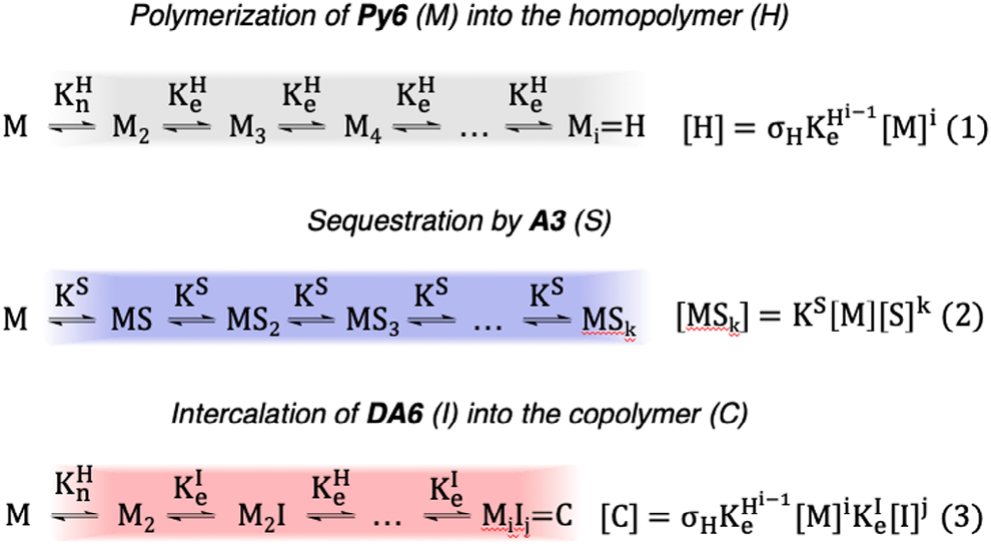
Equilibria describing the homo‐polymerization of **Py6** (M) into the homopolymer H (Equation 1), the sequestration by **A3** (S in Equation 2), and the co‐polymerization with **DA6** (I) based on the elongation from the nucleus of M_2_ into the copolymer C (Equation 3). *σ*_H_ corresponds to the cooperativity factor of the homo‐polymerization, the subscript *i* corresponds to the number of monomers M in a homopolymer or copolymer, the subscript *j* corresponds to the number of intercalators I in a copolymer and the subscript *k* corresponds to the number of sequestrators S bound to a single molecule of the monomer M. Branching paths involving sequestration from intermediate states and changes in the equilibrium constants upon increasing number of binding events have not been considered for clarity.

Further in‐depth control over the concerted supramolecular equilibria in binary and tertiary mixtures can be achieved by adjusting the concentration of **Py6** (Figures S19–S26). As these influences are rather complex we will discuss them one by one and rationalize the observed behavior based on the underlying mass balance equations according to the supramolecular polymerization equilibrium model by Goldstein and Stryer (Figure 6).^[^ ^43^, ^44^ ^]^

1) Influence on the sequestration ability of **A3**: Reducing the concentration of **Py6** to *c* = 2.5 × 10^−6^ m results in pronounced depolymerization in mixtures with **A3** (Figure S19f), while an increase in the concentration of **Py6** to *c* = 7.5 × 10^−6^ m results in a similar influence of the sequestrator compared to the previous reference at *c* = 5.0 × 10^−6^ m (Figure S20f). These observations can be rationalized by the fact that the concentration of the homopolymer of **Py6** with a length of *i* monomers depends on the concentration of **Py6** to the factor of *i* and the corresponding equilibrium constant *K*_e_^H^ (Figure 6, Equation 1). In contrast, the sequestration process is much more significantly influenced by the equilibrium constant of the sequestration (*K* ^S^) and the sequestrator concentration to the factor of *k*, where *k* corresponds to the number of binding events between **Py6** and its sequestrator (Figure 6, Equation 2). Accordingly, a decrease in the concentration of **Py6** results in a relative shift toward the sequestered monomer. It should be noted, that analogous reasoning applies to the influence of **A3** on the supramolecular polymerization of **DA6** (Figure S27, Discussion S2), which needs to likewise be taken into account in tertiary mixtures (vide infra).

2) Influence on the intercalation ability of **DA6**: In mixtures with **DA6** the spectroscopic signature of the homopolymer is drastically reduced with a decrease in the concentration of **Py6** and the copolymer with **DA6** becomes the most prevalent species even under relatively low additive concentrations (Figure S22a). In contrast, increased concentrations of **Py6** make the narcissistic assembly pathway more robust, even under higher additive loading (Figure S24b). This shift in equilibrium is also reflected in the two‐step transition in the heating curves, highlighting the more robust narcissistic pathway at low temperatures (Figure S24c,d). Again, the underlying mass balance equations serve as an explanation. The concentration of the heteropolymer of **Py6** and **DA6** with identical amounts of **Py6** depends on the concentration of **DA6** to the factor of *j* and the equilibrium constant *K*_e_^I^, where *j* likewise represents the number of **DA6** molecules in the heteropolymer (Figure 6, Equation 3). As this additional term is absent in the mass balance equation of the homopolymer (Figure 6, Equation 1) it becomes more influential upon decreasing the concentration of **Py6**.

3) Concentration influence in tertiary mixtures: In tertiary mixtures, all mass balance equations must be considered simultaneously, yet the relative trends observed in binary mixtures remain intact. Accordingly, a decrease in the concentration of **Py6** leads to a relative shift of the concerted equilibria toward the sequestered monomer as well as the heteropolymer with **DA6** (Figures S19, S20 for sequestered monomer; Figures S22, S23 for heteropolymer). To quantify this behavior, we evaluated the changes in the *T*_50_ value as a proxy for the melting temperature (see Materials and Method section in the Supporting Information). In the presence of **DA6**, a decrease in the sequestration ability of **A3** toward the homopolymer of **Py6** can be appreciated (Figure S26a). Additionally, since the stability of the heteropolymer depends on the concentration of **Py6** as well as **DA6** it can be stabilized by increasing the concentration of either building block (Figure S26b–d). However, while an increase in the concentration of **DA6** (Figures S22, S23) exclusively affects the heteropolymer pathway, an increase in the concentration of **Py6** simultaneously shifts the overall equilibrium toward the homopolymer (Figures S24, S25). Such complex concerted equilibria where a component is involved in multiple different supramolecular ensembles hint toward a potential dual use of individual supramolecular synthons in complex systems. Understanding these coordinated phenomena will provide the necessary framework to actualize the full potential of supramolecular systems chemistry.

## Conclusion

In summary, we have demonstrated that retrosynthetically analyzing a model supramolecular polymer (**Py6**) enables the rapid design of a variety of additive molecules with drastically different propensities to access concerted supramolecular equilibria and their corresponding assembly pathways. Namely, we found that the additive, which still had an identical assembly directing intermolecular binding moiety (**DA6**) was still able to act as an intercalator with **Py6**. However, due to the reduction in molecular similarity and the loss in binding strength, it was also found to act as a potent sequestrator at low concentrations. In contrast, **TB3** and **A3**, which only have one amide moiety in their molecular design, can only act as sequestrators, although their efficacy varies based on the size of the conjugated *π*‐skeleton, providing a clear structure‐property relationship. Further, we found that even when **TB3** is present at concentrations that induce its assembly, it still cannot act as an intercalator with **Py6** emphasizing the relevance of enthalpic penalties when engineering monomers intended for intercalation/sequestration. Based on these insights, we showed that our design approach offers access to dilution‐induced supramolecular polymerization, an intriguing property, that has been rarely observed in the literature. What's more, tertiary mixtures of **Py6**, **DA6**, and **A3** can exhibit either dual sequestration or a hybrid system, in which an intercalator and a sequestrator compete for the interaction with **Py6**. Particularly the unprecedented competitive hybrid system of an intercalator and a sequestrator holds important insight for complex supramolecular systems. For instance, it could be demonstrated that by carefully fine‐tuning the concentration of the different species a specific supramolecular synthon (**Py6**) can be stabilized within more than one supramolecular ensemble (homopolymer and heteropolymer with **DA6**). These results demonstrate, that supramolecular synthons can fulfill more than one role in complex supramolecular systems, provided that the different concerted equilibria are properly accounted for.^[^ ^18^, ^25^, ^45^, ^46^ ^]^ We anticipate that the results disclosed herein can contribute to the development of complex, yet robust supramolecular systems. Additionally, the clear design principle required for intercalation offers the potential for more in‐depth control of social self‐sorting interactions and their associated photophysical phenomena. Given the fact that **DA6** doesn't engage in any significant aromatic interactions with the chromophore of **Py6**, this hints at the possibility of inducing supramolecular spacers within an ensemble where long‐range interactions between chromophores can be more carefully controlled. Work to exploit such fine control of interchromophore interactions is currently underway in our laboratory.

## Supporting Information

The authors have cited additional references within the Supporting Information.^[^ ^47^, ^48^, ^49^, ^50^ ^]^

## Note

A previous version of this manuscript has been deposited on a preprint server.^[^ ^51^ ^]^

## Conflict of Interests

The authors declare no conflict of interest.

## Supporting information



Supporting Information

## Data Availability

The data that support the findings of this study are available in the supplementary material of this article.
