# Supplementary material for: Elucidating the Boundary of Intercalation vs Sequestration in Supramolecular Polymers by Retrosynthetic Design Toward the Construction of Complex Supramolecular Systems
Source: Angew Chem Int Ed Engl. 2025 Mar 10;64(19):e202501693. doi: 10.1002/anie.202501693 (PMC12051726; doi:10.1002/anie.202501693)
Supplement: Supplementary file 1 — Supporting Information [file ANIE-64-e202501693-s001.pdf]

Supporting Information
©Wiley-VCH 2021
69451 Weinheim, Germany

Elucidating the Boundary of Intercalation *vs.* Sequestration in Supramolecular Polymers by Retrosynthetic Design Towards the Construction of Complex Supramolecular Systems

Nils Bäumer,*[a] Soichiro Ogi *[b] and Shigehiro Yamaguchi [a,b,c]

Abstract: Controlled social self-sorting by intercalation can offer distinct properties at the supramolecular level that go beyond the sum of its parts. Likewise, controlling narcissistic self-sorting by sequestration can induce unique system properties. In contrast, the interface between the two cases has hitherto remained underexplored, and clear design rules remain elusive. Herein it is demonstrated that by fine-tuning the molecular similarity of supramolecular synthons, intricate control over concerted supramolecular equilibria can be achieved. By reducing the molecular similarity, a former intercalator can be tuned to become a strong or weak sequestrator. Understanding these roles in binary mixtures allows to rationalize more complex tertiary systems. Consequently, the influence of an uncommon dual sequestration mechanism is revealed. Further, an unprecedented hybrid mechanism between supramolecular intercalation and sequestration can be demonstrated. We are hopeful that the results presented herein will contribute to the development and understanding of concerted processes in complex supramolecular systems.

DOI: 10.1002/anie.2024XXXXX

Table of Contents

Materials and Methods.......... S3
Synthesis and Characterization S4
Additional spectroscopy S7
References.......... S32
NMR spectroscopy.......... S33

Materials and Methods

Characterization: ^1H and ^{13}C NMR spectra were recorded with a JEOL AL-400 spectrometer (400 MHz for ^1H, 100 MHz for ^{13}C) or a JEOL JNM-ECS400 (400 MHz for ^1H, 100 MHz for ^{13}C) in $CDCl_3$. The chemical shifts in ^1H NMR spectra are reported in δ ppm using the residual proton of the solvent as an internal standard ($CHCl_3$ δ 7.26), and those in ^{13}C NMR spectra are reported using the solvent signal as an internal standard ($CDCl_3$ δ 77.16). Mass spectra were measured with a Thermo Fisher Scientific Exactive spectrometer with the ESI ionization method.

Synthesis: All reactions were performed with dry glassware and under a nitrogen atmosphere unless stated otherwise. Thin layer chromatography (TLC) was performed on glass plates coated with 0.25 mm thickness of silica gel $60F_{254}$ (Merck). Column chromatography was performed in self-packed columns using silica gel PSQ100B (Fuji Silysia Chemicals). Preparative Gel permeation Chromatography (GPC) was performed using LC-918 (Japan Analytical Industry) equipped with gel column (JAIGEL-2.5H and -3H) using $CHCl_3$ as eluent. All chemicals were purchased from commercial suppliers and used without further purification. Anhydrous THF was purchased from Kanto Chemicals and further purified by Glass Contour Solvent Systems.

UV-vis spectroscopy: The spectroscopic measurements were conducted under ambient conditions using solvents of spectroscopic grade. UV-vis absorption spectra were recorded using quartz cuvettes of 1 cm path length with a JASCO V-750 and a V-770 spectrophotometer equipped with a JASCO ETCR-762 cell holder for temperature control.

Dynamic light scattering: DLS was performed with a Zetasizer Nano particle analyser ZEN 3600 (Malvern) equipped with a 4 mW 632.8 nm laser using a 90° detector angle. Measurements were performed using quartz cuvettes of 1 cm path length.

Transmission electron microscopy: TEM was performed with a JEM-1400EM (JEOL) using an acceleration voltage of 80 kV. The samples (10 µL) were drop-casted on a carbon-coat copper grid (400 mesh) and the solvent was removed with a filter paper, followed by drying under reduced pressure. The measurements were conducted without additional staining.

Fourier-transform infrared spectroscopy: FT-IR was performed on a JACSO FT-IR-4200 spectrometer.

Sample preparation: The compounds were dissolved in a stock solution at a high concentration (> 5.0×10^{-4} M) in chloroform. Afterwards the necessary amounts of the stock solution were transferred to a screw cap vial and the solvent was evaporated using a nitrogen stream followed by treatment *in vacuo* to complete dryness using a Schlenk line. To prepare the measurement solutions the necessary amount of solvent was added and the screw cap vial was heated using a heatgun to dissolve the compound. The measurement solutions were then transferred to the cuvettes immediately prior to measurement. For temperature-dependent measurements the cuvettes were additionally sealed shut using a Teflon tape. Samples for co-assembly studies were prepared in the same fashion by preparing separated stock solutions of both compounds and transferring them to the same vial prior to evaporation. Samples using high concentrations of additives, such as **A3** were prepared by adding the stock solution of other compounds directly to the solid sample of **A3**, followed by co-dissolution in chloroform and subsequent evaporation and drying. Deviating sample preparation protocols are detailed in the specific figure captions.

Calculation of T_{50}: To calculate the temperature, at which the temperature-dependent relative changes in the extinction coefficient have reached half their value at T = 293 K (T_{50}) two values have been used as fixed references to allow for better comparability in mixed systems where more than one supramolecular entity is present. For the homopolymer of **Py6** that value corresponds to the first data point obtained for **Py6** in isolation as a concentration of c = 5.0×10^{-6} M. For the copolymer of **Py6** and **DA6** the first data point obtained for a mixture of **Py6** at c = 2.5×10^{-6} M and **DA6** at c = 2.0×10^{-5} M.

Synthesis and Characterization

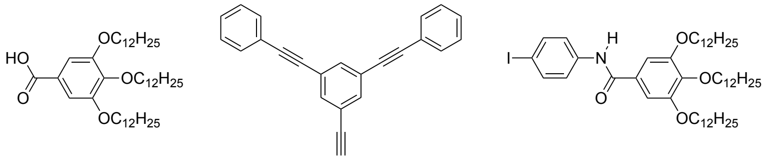

1 **2** **3**

Scheme S1. Chemical structures of compounds **1**, **2** and **3**.

Compounds **1**[1], **2**[2] and **3**[3] were synthesized according to previously reported procedures and showed identical properties to those reported therein.

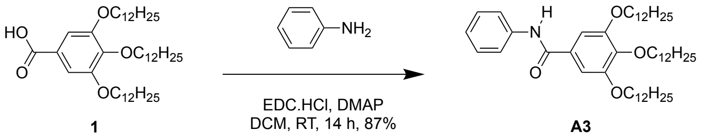

Scheme S2. Synthetic scheme for the preparation of **A3**.

Compound **A3**

Compound **1** (675 mg, 1.00 mmol, 1.00 eq.), 1-ethyl-3-(3-dimethylaminopropyl)carbodiimide (EDC)·HCl (200 mg, 1.04 mmol, 1.04 eq.) and dimethylaminopyridine (DMAP) (130 mg, 1.06 mmol, 1.06 eq.) were dissolved in dried dichloromethane (DCM) (40 mL) and stirred at room temperature for 120 min. Afterwards aniline (0.100 mL, 102 mg 1.10 mmol, 1.10 eq) was added and the reaction mixture was stirred at room temperature for 14 h. The reaction mixture was washed with a ½ sat. NaCl aqueous solution (3 × 20 mL) and dried over Na_2SO_4. The solvent was removed under reduced pressure and the crude product was purified by column chromatography (SiO_2; 1/1 hexane/DCM to DCM, R_f = 0.10 for 1/1 hexane/DCM, R_f = 0.73 for DCM) to give the target compound as a white solid.

Yield: 652 mg, 0.869 mmol, 87%.

Mp: 63–64 °C; ^{1}H NMR (400 MHz, 298 K, $CDCl_3$): δ 7.92 (s, 1H); 7.65–7.62 (m, 2H); 7.37–7.33 (m, 2H); 7.16–7.11 (m, 1H); 7.03 (s, 2H); 4.02–3.96 (m, 6H); 1.83–1.72 (m, 6H); 1.50–1.42 (m, 6H); 1.34–1.24 (m, 48H); 0.91–0.86 (m, 9H); ^{13}C NMR (100 MHz, 298 K, $CDCl_3$): δ 165.9, 153.3, 141.5, 138.2, 130.0, 129.2, 124.6, 120.3, 105.9, 73.7, 69.5, 32.1, 30.5, 29.9, 29.84, 29.78, 29.7, 29.54, 29.50, 29.47, 26.2, 22.8, 14.3; HRMS (ESI, positive): *m*/*z* calcd. for $C_{49}H_{84}NO_4$: 750.6400 ($[M+H]^+$), found: 750.6398.

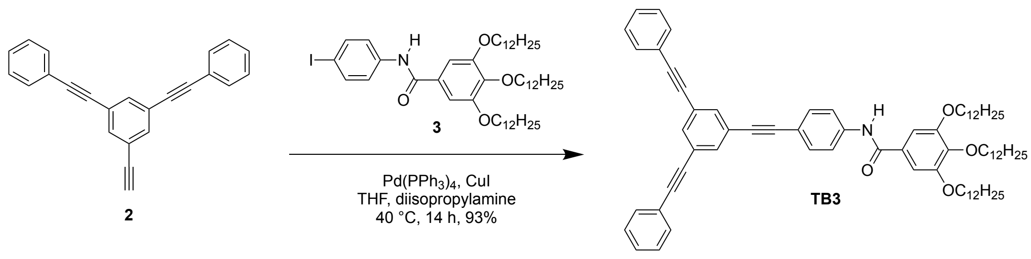

Scheme S3. Synthetic scheme for the preparation of **TB3**.

Compound **TB3**

Compound **3** (175 mg, 0.200 mmol, 1.0 eq.), CuI (2 mg, 0.01 mmol, 5 mol%) and $Pd(PPh_3)_4$ (20 mg, 0.017 mmol, 8 mol%) were dissolved in mixture of dried tetrahydrofuran (THF, 20 mL) and diisopropylamine (10 mL) under a nitrogen atmosphere and stirred at room temperature for 45 min. Afterwards compound **2** (60 mg, 0.20 mmol, 1.0 eq) was added and the reaction mixture was stirred at 40 °C for 14 h. Afterward, the reaction mixture was cooled to room temperature and the solvent was removed under reduced pressure. The crude product was purified by column chromatography (SiO_2; hexane to 1/1 hexane/DCM, R_f = 0.15 for 1/1 hexane/DCM) to give the target compound as a white solid.

Yield: 195 mg, 0.185 mmol, 93%.

Mp: 107–108 °C; ^{1}H NMR (400 MHz, 298 K, $CDCl_3$): δ 8.16 (s, 1H), 7.73–7.67 (m, 2H); 7.67–7.62 (m, 3H); 7.58–7.50 (m, 6H); 7.40–7.33 (m, 6H); 7.02 (s, 2H); 4.02 (t, J = 6.6 Hz, 2H); 3.96 (t, J = 6.5 Hz, 4H); 1.83–1.73 (m, 6H); 1.51–1.42 (m, 6H); 1.36–1.24 (m, 48H); 0.92–0.87 (m, 9H); ^{13}C NMR (100 MHz, 298 K, $CDCl_3$): δ = 166.0, 153.3, 141.5, 138.6, 134.1, 132.7, 131.8, 129.7, 128.7, 128.5, 124.20, 124.15, 122.9, 120.0, 118.6, 105.8, 90.6, 90.5, 88.0, 87.8, 73.7, 69.5, 32.1, 30.5, 29.9, 29.8, 29.7, 29.6, 29.52, 29.48, 26.2, 22.8, 14.3; HRMS (ESI, positive): m/z calcd. for $C_{73}H_{95}NO_4Na$: 1072.7159 ($[M+Na]^+$); found: 1072.7156.

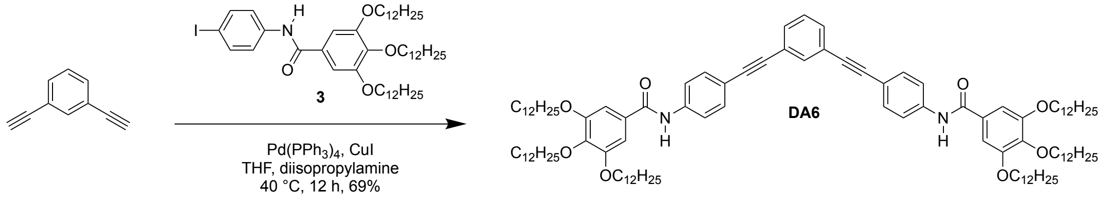

Scheme S4. Synthetic scheme for the preparation of **DA6**.

Compound **DA6**

Compound **3** (350 mg, 0.399 mmol, 2.0 eq.), CuI (4 mg, 0.02 mmol, 10 mol%) and $Pd(PPh_3)_4$ (40 mg, 0.035 mmol, 17 mol%) were dissolved in mixture of dried THF (40 mL) and diisopropylamine 20 mL under a nitrogen atmosphere and stirred at room temperature for 30 min. Afterwards 1,3-diethynylbenzene (28.4 µL, 25 mg, 0.20 mmol, 1.0 eq) was added and the reaction mixture was stirred at 40 °C for 12 h. Afterward, the reaction mixture was cooled to room temperature and the solvent was removed under reduced pressure. The crude product was purified by column chromatography (SiO_2; hexane to 1/1 hexane/DCM, R_f = 0.05 for 1/1 hexane/DCM, R_f = 0.75 for DCM) to give the target compound as a white solid.

Yield: 223 mg, 0.137 mmol, 69%.

Mp: 131–132 °C; ^{1}H NMR (400 MHz, 298 K, $CDCl_3$): δ 8.02 (s, 2H); 7.71–7.69 (m, 1H); 7.69–7.65 (m, 4H); 7.54–7.51 (m, 4H); 7.49–7.46 (m, 2H); 7.35–7.30 (m, 1H); 7.02 (s, 4H); 4.02–3.96 (m, 12H); 1.83–1.72 (m, 12H); 1.50–1.42 (m, 12H); 1.33–1.24 (m, 96H); 0.90–0.86 (m, 18H); ^{13}C NMR (100 MHz, 298 K, $CDCl_3$): δ = 165.9, 153.3, 141.6, 138.4, 134.6, 132.6, 131.3, 129.8, 128.6, 123.8, 119.9, 118.9, 105.8, 89.9, 88.5, 73.7, 69.5, 32.1, 30.5, 29.90, 29.85, 29.8, 29.7, 29.6, 29.54, 29.51, 29.48, 26.2, 22.8, 14.3; HRMS (ESI, positive): *m*/*z* calcd. for $C_{108}H_{169}N_2O_8$: 1622.2879 ([*M*+H]$^+$); found: 1622.2853

Additional spectroscopy

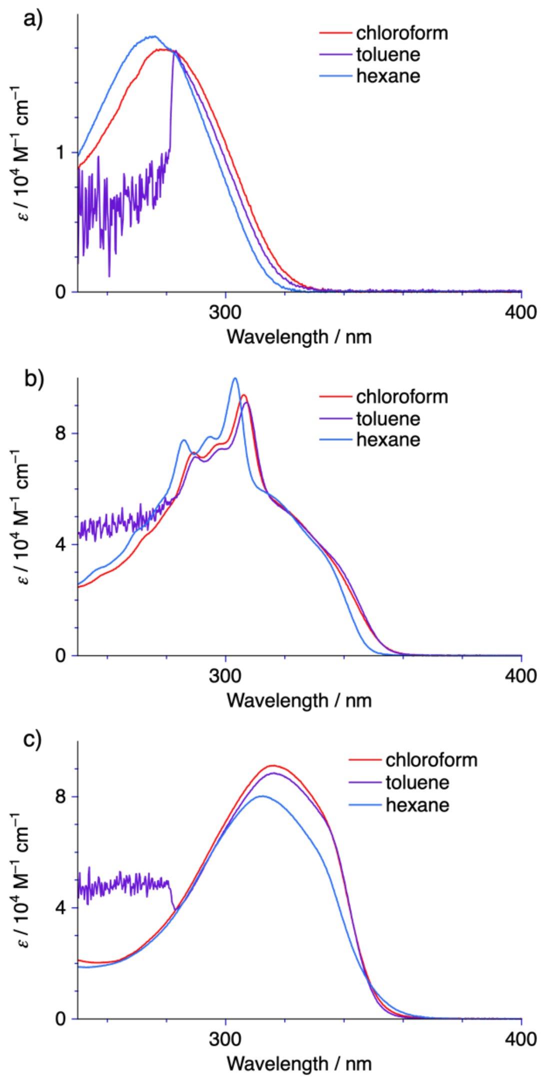

Figure S1. a-c) Solvent-dependent UV absorption spectra of **A3** (a), **TB3** (b), and **DA6** (c) at $c = 1.0 \times 10^{-5}$ M at $T = 293$ K.

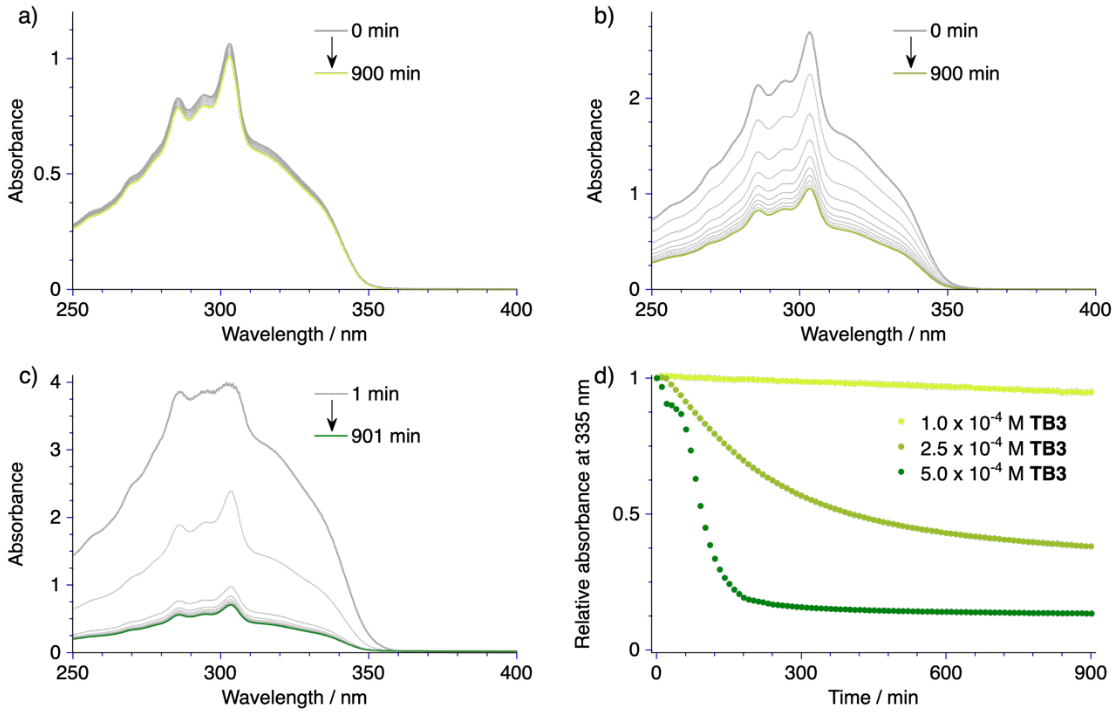

Figure S2. a-c) Time-dependent UV absorption spectra of **TB3** at $c = 1.0 \times 10^{-4}$ M (a), $c = 2.5 \times 10^{-4}$ M (b), and 5.0×10^{-4} M (c) in hexane at $T = 293$ K after heating to 343 K for 15 minutes followed by transfer to a pre-cooled UV spectrophotometer. d) Relative changes in absorbance determined at $\lambda_{abs} = 335$ nm plotted against the time. The changes in absorption as well as the secondary plot are a consequence of the precipitation of the polymers of **TB3**, which were found to be highly insoluble, and should thus be interpreted as a proxy for the time-dependent assembly process.

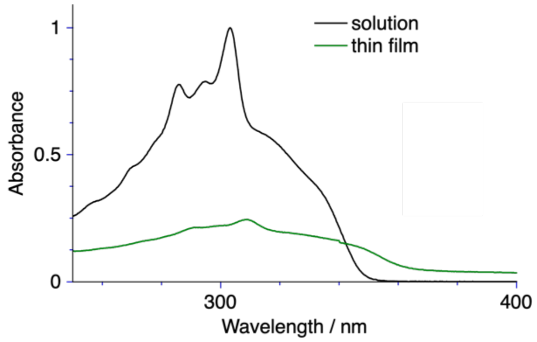

Figure S3. Solution and thin film UV-vis absorption spectra of **TB3**. The solution sample was recorded at $c = 1.0 \times 10^{-5}$ M at $T = 293$ K in hexane and the thin film sample was prepared by dropcasting a small amount ($V = 20$ μL) of a hexane solution at $c = 5.0 \times 10^{-4}$ M immediately after heating to 343 K for 15 minutes to prevent the formation of precipitates.

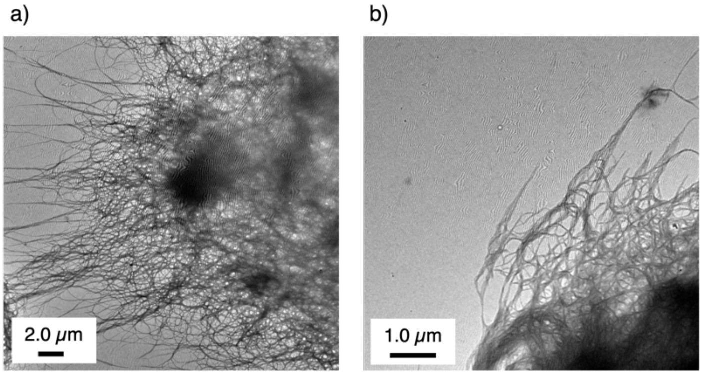

Figure S4. a,b) TEM micrographs obtained from drop-casting (V = 10 μL) a hexane solution of **TB3** (c = 5.0 × 10^{-4} M) after precipitation occurred.

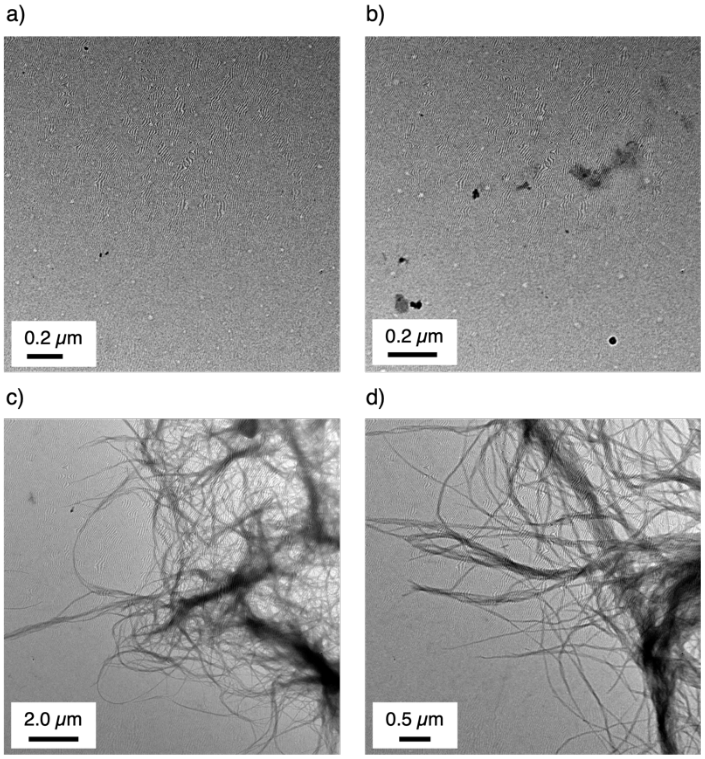

Figure S5. a-d) TEM micrographs obtained from dropcasting (V = 10 μL) a hexane solution of **DA6** (c = 2.0 × 10^{-5} M (a,b); 5.0 × 10^{-4} M (c,d)).

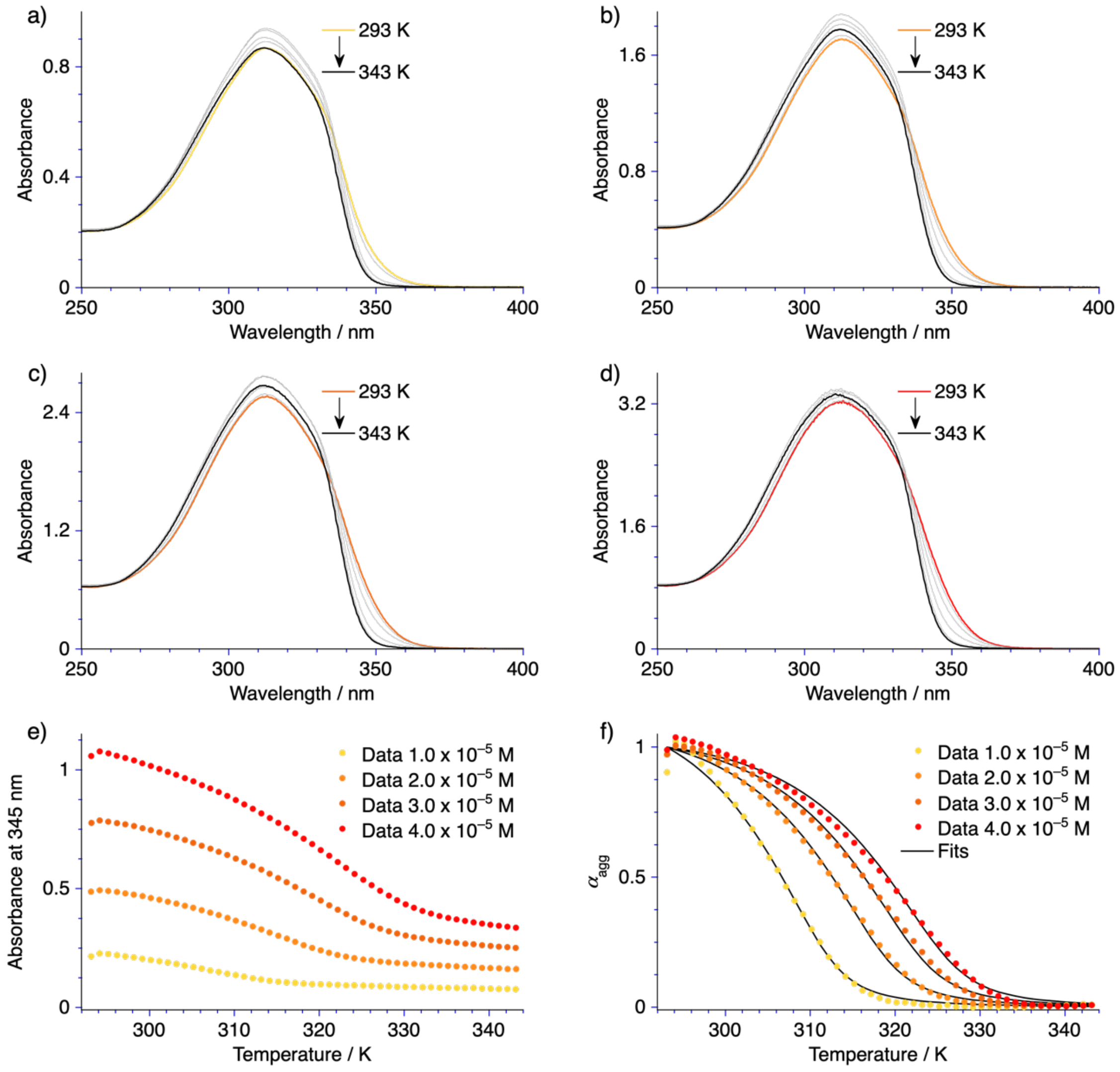

Figure S6. a-d) Temperature-dependent UV absorption spectra of **DA6** at $c = 1.0 \times 10^{-5}$ M (a); 2.0×10^{-5} M (b); 3.0×10^{-5} M (c) and 4.0×10^{-5} M (d) in hexane between $T = 293$ and 343 K using a heating rate of 1 K/min and a data interval of 1 K. e,f) Changes in absorbance at λ_{abs} = 345 nm (e) and changes in the aggregation parameter (α_{agg}) determined at λ_{abs} = 345 nm with fits to the nucleation elongation model (f) plotted against the temperature. Fits were obtained using a global fitting approach.[4]

The elongation enthalpy (ΔH_e^0), nucleation penalty (ΔH_n^0), entropy (ΔS^0) and elongation temperature (T_e) were obtained directly from the fitting procedure,[4] while the elongation (K_e) and nucleation (K_n) equilibrium constants, and degree of cooperativity (σ) were calculated using equation 1–3:

$$K_n = e\left(\frac{-\left((\Delta H_e - \Delta H_n) - T_e \Delta S\right)}{RT_e}\right) \; (1)$$

$$K_e = e\left(\frac{-(\Delta H_e - T_e \Delta S)}{RT_e}\right) \; (2)$$

$$\sigma = \frac{K_n}{K_e} \; (3)$$

Table S1. Thermodynamic parameters derived from fitting the heating curves in Fig. S6 to the nucleation-elongation model by ten Eikelder and co-workers using a global fitting approach.[4]

c / 10^{-5} M	ΔH_e (SD) / kJ mol^{-1}	ΔH_n (SD) / kJ mol^{-1}	ΔS (SD) / kJ mol^{-1} K^{-1}	ΔG^{0a} / kJ mol^{-1}	T_e (SD) / K	K_e / 10^4 M^{-1}	K_n / 10^2 M^{-1}	σ / 10^{-2}
1.0	–83.8 (1.1)	–10.0 (0.2)	–0.174 (0.004)	–31.8	310.3 (0.1)	10.0	20.9	2.1
2.0					317.1 (0.1)	5.0	11.4	2.3
3.0					321.2 (0.2)	3.3	7.9	2.4
4.0					324.2 (0.2)	2.5	6.2	2.5

[a]The standard Gibbs free energy was calculated for a temperature of T = 298 K.

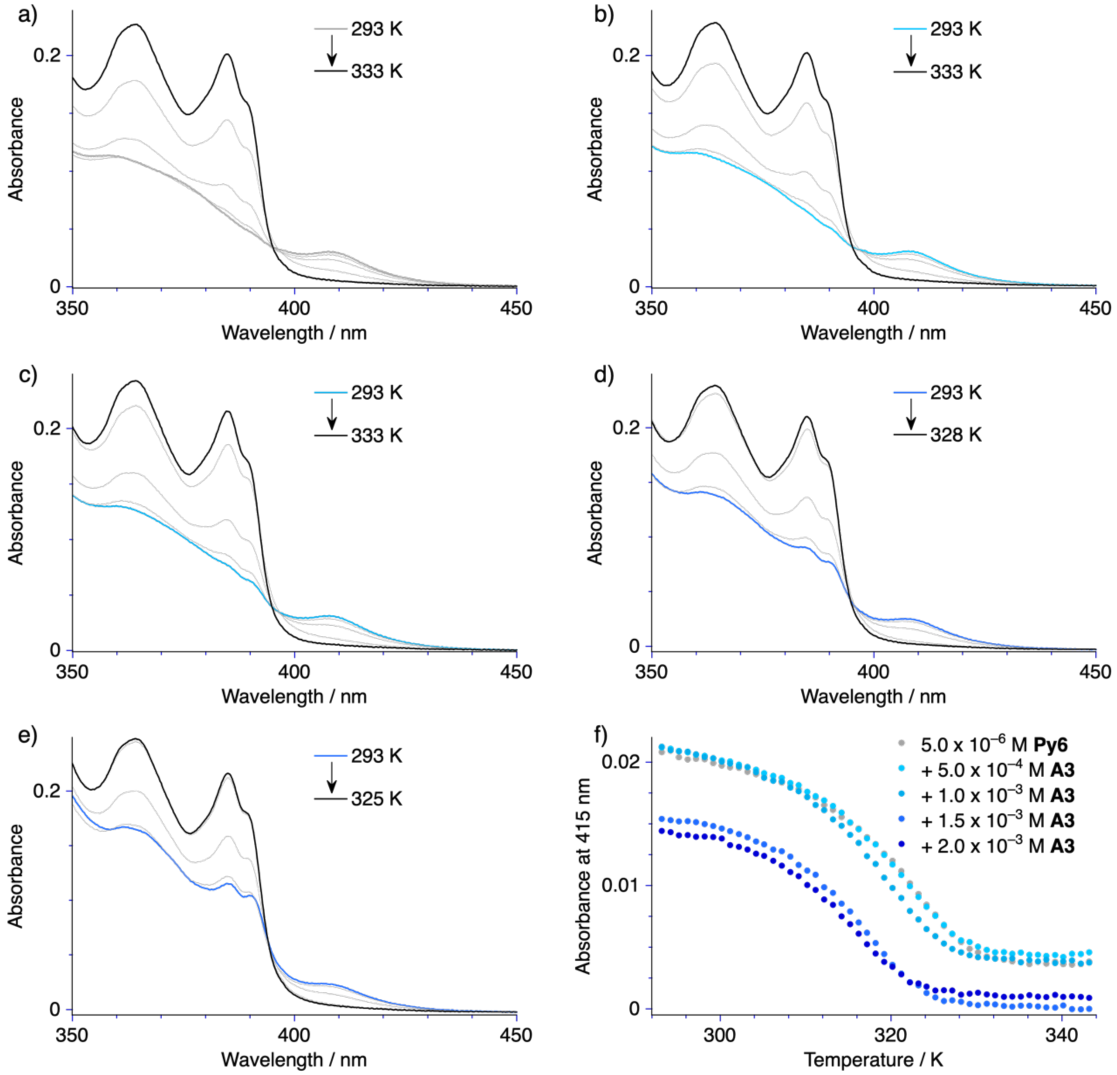

Figure S7. a-e) Temperature-dependent UV-vis absorption spectra of **Py6** (c = 5.0 × 10^{-6} M) in hexane between T = 293 and 333 K using a heating rate of 1 K/min and a data interval of 1 K in isolation (a) as well as in the presence of **A3** at c = 5.0 × 10^{-4} M (b); 1.0 × 10^{-3} M (c); 1.5 × 10^{-3} M (d); 2.0 × 10^{-3} M (e). f) Changes in absorbance at λ_{abs} = 415 nm plotted against the temperature.

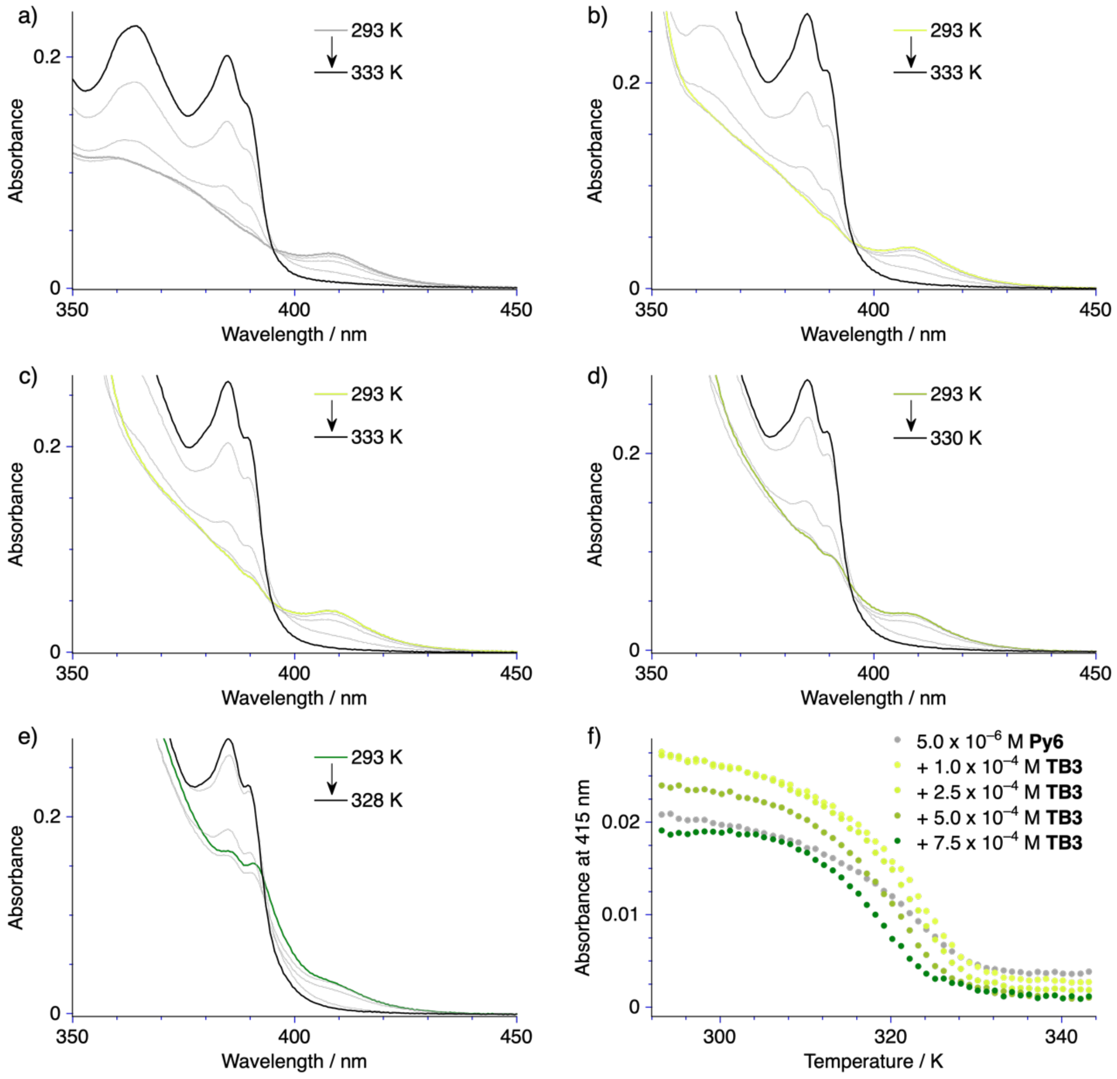

Figure S8. a-e) Temperature-dependent UV-vis absorption spectra of **Py6** (c = 5.0 × 10^{-6} M) in hexane between T = 293 and 333 K using a heating rate of 1 K/min and a data interval of 1 K in isolation (a) as well as in the presence of **TB3** at c = 1.0 × 10^{-4} M (b); 2.5 × 10^{-4} M (c); 5.0 × 10^{-4} M (d); 7.5 × 10^{-4} M (e). f) Changes in absorbance at λ_{abs} = 415 nm plotted against the temperature.

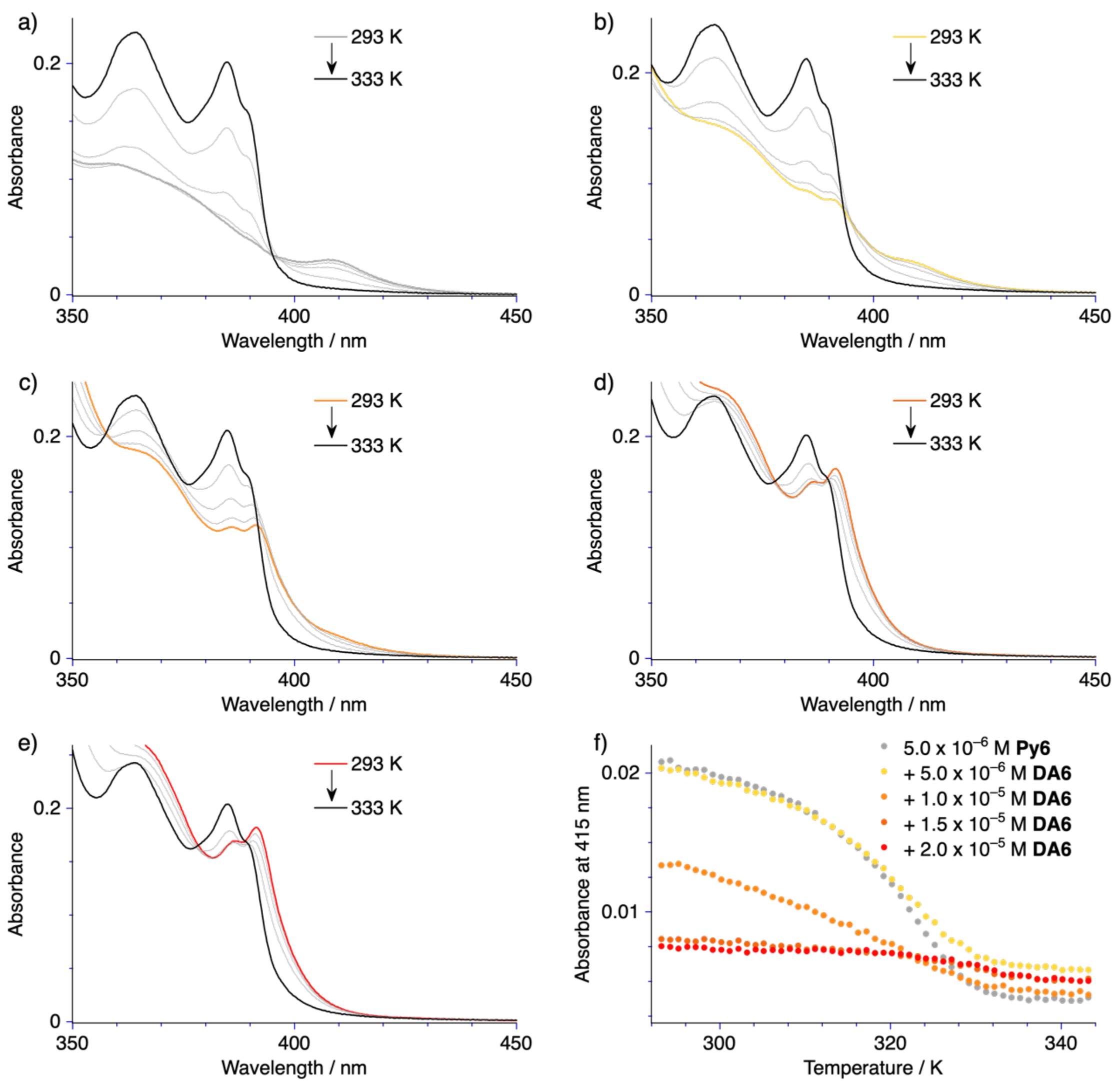

Figure S9. a-e) Temperature-dependent UV-vis absorption spectra of **Py6** ($c = 5.0 \times 10^{-6}$ M) in hexane between T = 293 and 333 K using a heating rate of 1 K/min and a data interval of 1 K in isolation (a) as well as in the presence of **DA6** at $c = 5.0 \times 10^{-6}$ M (b); 1.0×10^{-5} M (c); 1.5×10^{-5} M (d); 2.0×10^{-5} M (e). f) Changes in absorbance at λ_{abs} = 415 nm plotted against the temperature.

Table S2. Changes in the elongation temperature (ΔT_e) of the homopolymer of **Py6** relative to reference experiments of **Py6** in isolation ($c = 5.0 \times 10^{-6}$ M, T_e = 327.0 K) with the added equivalents of the additive given in brackets.

Additive	ΔT_e / K (additive loading / eq.)			
**A3**	–1.3 (100)	–3.7 (200)	–6.9 (300)	–8.5 (400)
**TB3**	+0.2 (20)	–0.5 (50)	–3.7 (100)	–7.8 (150)
**DA6**	–0.6 (1)	– (2)[a]	– (3)[a]	– (4)[a]

[a] Under these conditions the system is partially or fully dominated by the heteropolymer.

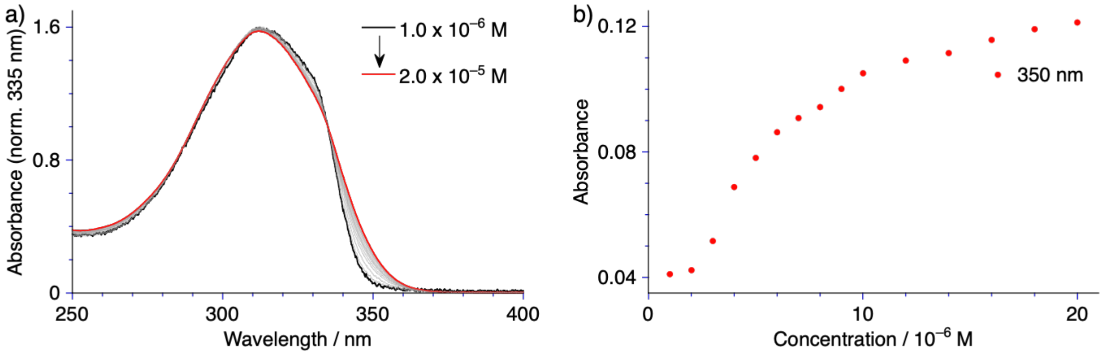

Figure S10. a) Normalized concentration-dependent UV-vis absorption spectra of **DA6** in hexane at T = 293 K. b) Changes in absorbance at λ_{abs} = 350 nm extracted from the normalized spectra in (a) plotted against the concentration of **DA6**.

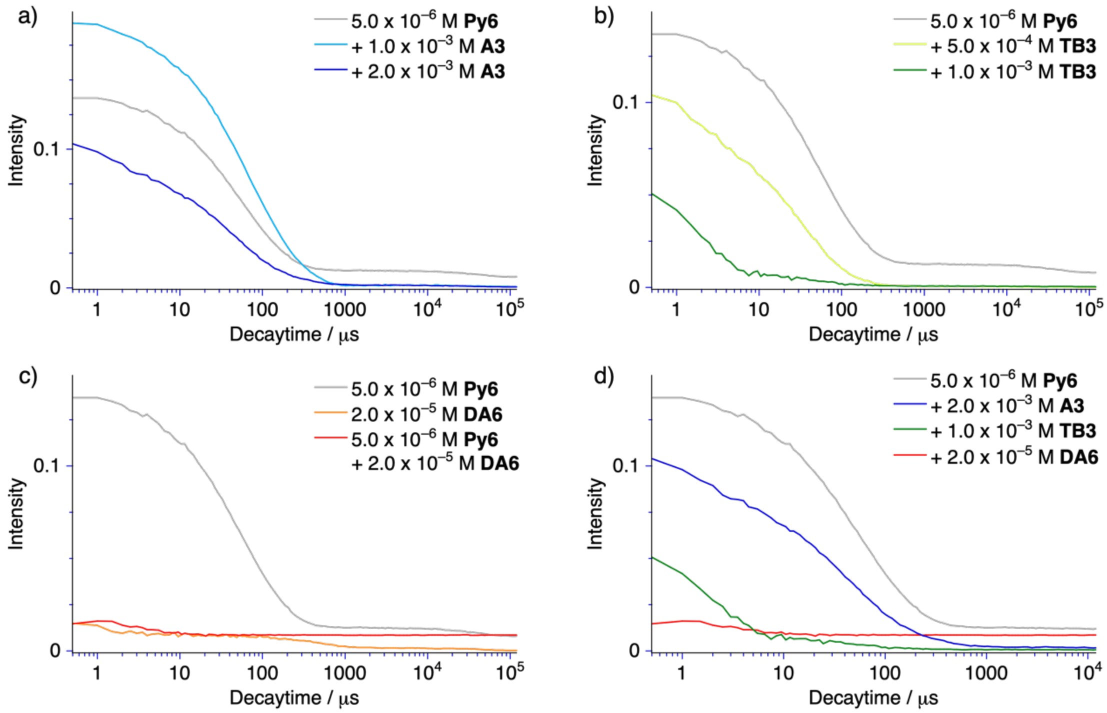

Figure S11. a-c) DLS correlation functions of **Py6** (c = 5.0 × 10^{-6} M) in hexane at T = 298 K in the presence of increasing amounts of **A3** (a), **TB3** (b) and **DA6** (c). d) DLS correlation functions with the highest presence of additive from (a-c) shown for direct comparison.

Supplementary discussion 1

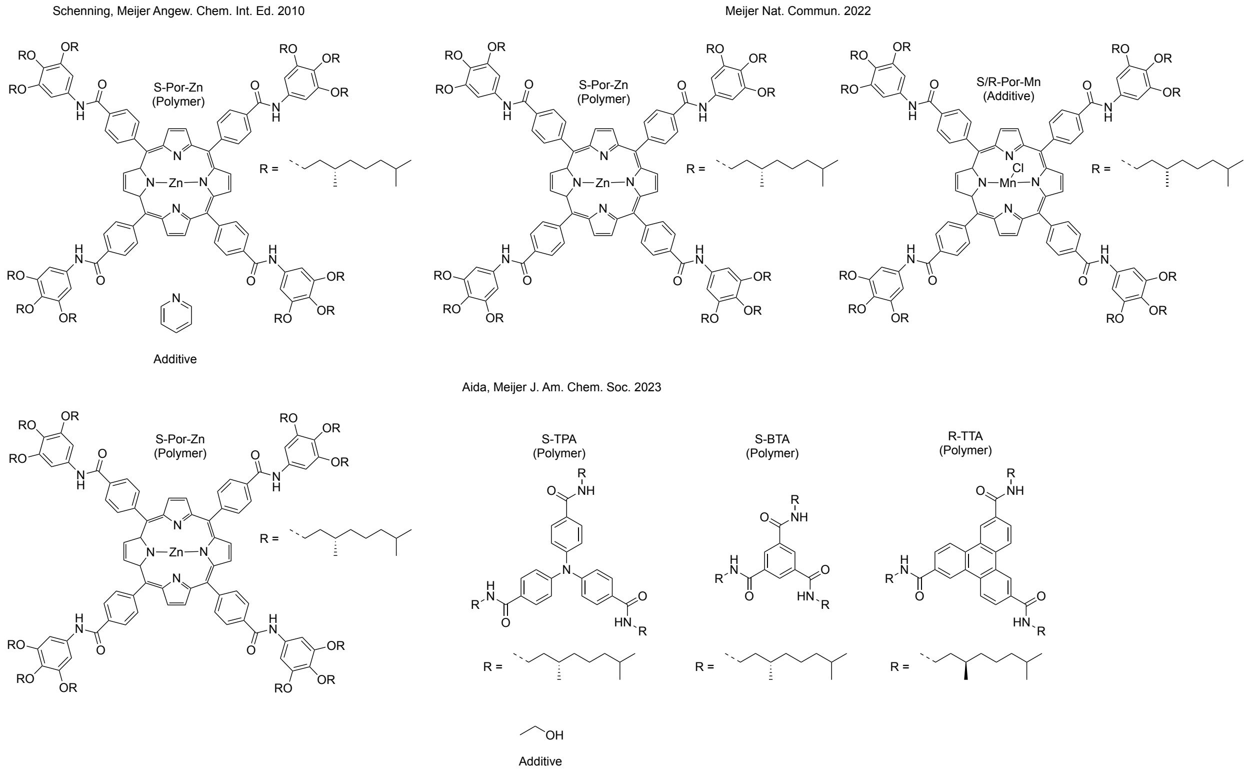

Figure S12. Molecular structures of the reported monomers and corresponding additives employed for dilution-induced supramolecular polymerization.

In their seminal work on dilution-induced supramolecular polymerization *Meijer* and coworkers focused on a Zn-containing porphyrin derivative (S-Por-Zn), which showed phase transitions from a dimeric species that is coordinated on either side by the porphyrin additive to the polymerized state upon dilution (Figure S12).[5] In their analysis they highlighted a key parameter making their system at hand ideal to enforce dilution-induced supramolecular polymerization. Namely, S-Por-Zn shows a high degree of cooperativity ($\sigma = 5.0 \times 10^{-5}$). Consequently, relatively large quantities of the molecularly dissolved state co-exist in equilibrium with the polymer. Subsequent modeling highlighted, that this monomer fraction is key to achieve the complexation process, which ultimately enables the dilution-induced supramolecular polymerization.[5]

In their subsequent study utilizing the manganese containing derivative as an additive they similarly observed dilution induced supramolecular polymerization. However, in this case the supramolecular equilibrium was shifted between short oligomers of the Mn-appended stack (H- and J-type aggregate) as well as the elongated H-type homopolymer of S-Por-Zn.[6]

From a qualitative viewpoint the investigated binary mixture **Py6** and **A3** can be considered to be similar to that of S-Por-Zn and S/R-Por-Mn, where **A3** (like S/R-Por-Mn) is unable to engage in elongation making the interactions with **Py6** more favorable the higher the concentration becomes, effectively sequestering **Py6** in concerted equilibria.

Following that the groups of *Aida* and *Meijer* investigated assembly landscapes including dilution induced polymerization and biphasic phase transitions using ethanol as additive in more detail.[7] Their analysis showed drastic differences between their earlier model compound S-Por-Zn and other supramolecular polymers stabilized by threefold hydrogen bonding. Namely, in the case of S-Por-Zn (4-fold hydrogen bonding, high degree of cooperativity) the dilution-induced polymerization has a very narrow transition, whereas in the case of S-TPA (threefold hydrogen bonding, $\sigma \approx 1.7 \times 10^{-4}$) this transition occurs over a wider concentration range under identical additive conditions (compare Figure 4c and Figure S7d in reference S7).[7,8]

By comparing these supramolecular model compounds to **Py6** some indications about their behavior upon dilution can be extracted. **Py6** exhibits a relatively modest degree of cooperativity ($\sigma = 4.0 \times 10^{-2}$) with an even more negative Gibbs free energy than S-TPA (–40.7 kJ/mol for **Py6** and –31.6 kJ/mol for S-TPA at $T = 298$ K). This means that the effective concentration of the monomer of **Py6** (which was found to be crucial for dilution-induced polymerization processes),[5] will be significantly lower than the one of previously reported model compounds. Consequently, in our system the overall spectral changes are rather modest and the complete transition cannot be observed, making the exact determination of the binding constants unfeasible.

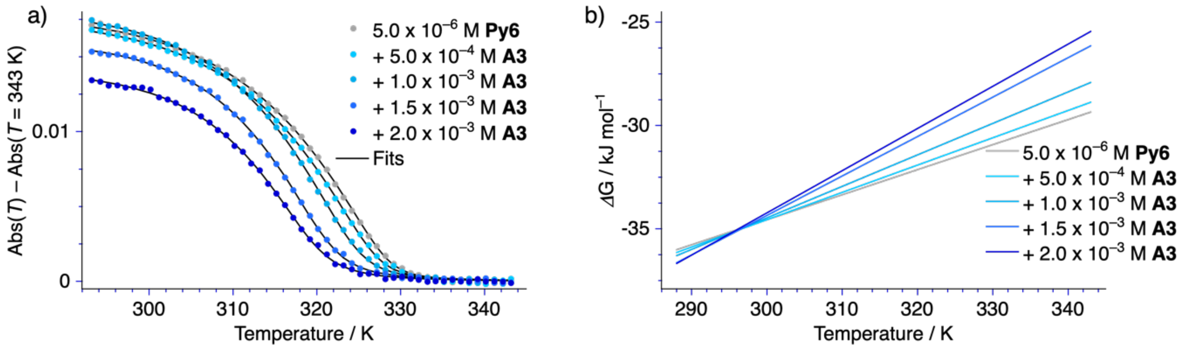

Figure S13. a) Temperature-dependent changes in absorbance at λ_{abs} = 415 nm of the UV-vis absorption spectra shown in Figure S7 relative to the absorbance at T = 343 K with fits to the nucleation elongation model plotted against the temperature. Fits were obtained using an individual fitting approach.[4] b) Changes in the Gibbs free energy of the homopolymer of **Py6** plotted against the temperature with increasing amounts of **A3** derived from the fits shown in (a). The absolute values should be interpreted with care as a qualitative testament to the decrease in stability induced by the sequestration by **A3**, rather than a quantitative measure.

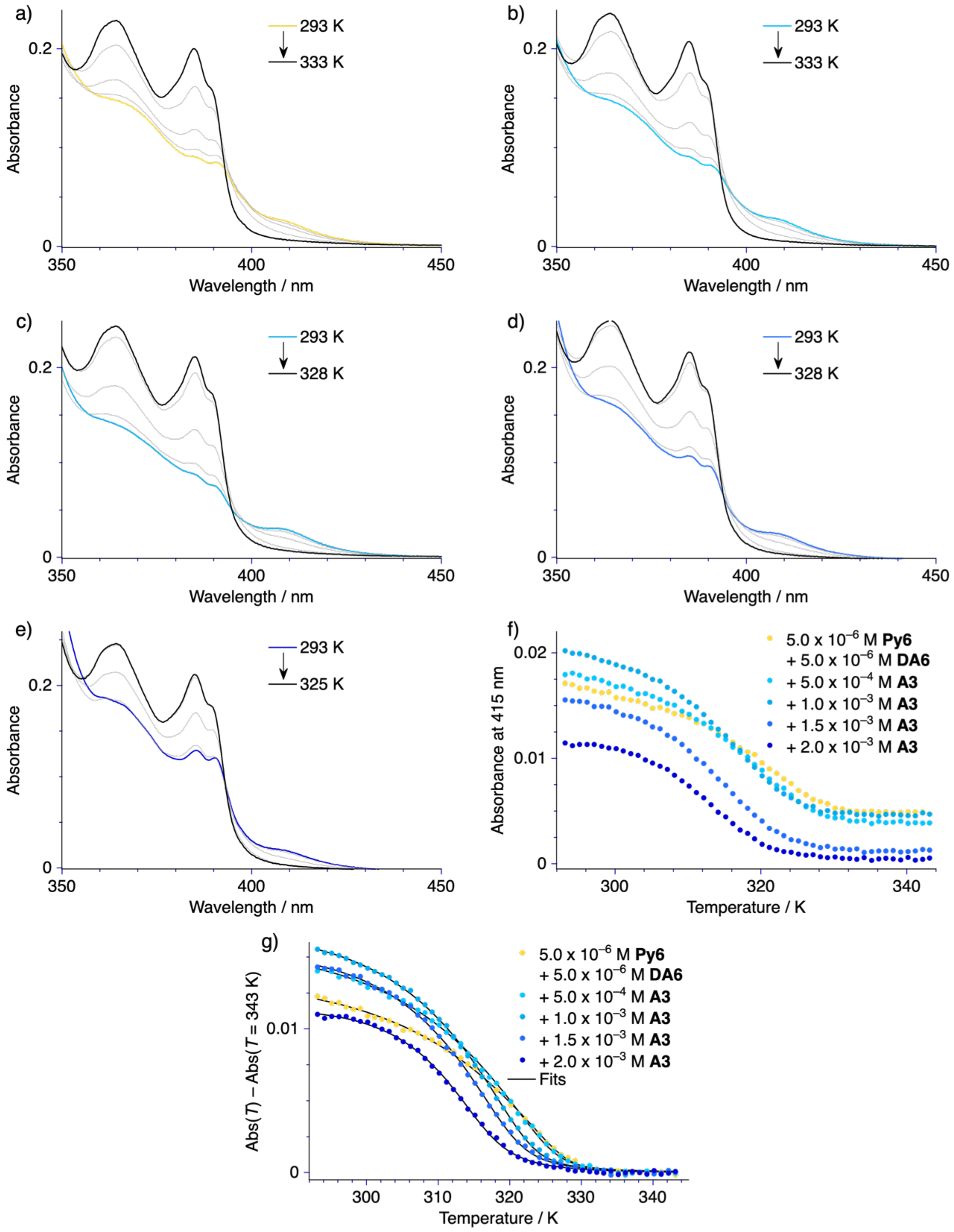

Figure S14. a-e) Temperature-dependent UV-vis absorption spectra of **Py6** (c = 5.0 × 10^{-6} M) and **DA6** (c = 5.0 × 10^{-6} M) in hexane between T = 293 and 333 K using a heating rate of 1 K/min and a data interval of 1 K in a binary mixture (a) as well as in tertiary mixtures with **A3** at c = 5.0 × 10^{-4} M (b); 1.0 × 10^{-3} M (c); 1.5 × 10^{-3} M (d); 2.0 × 10^{-3} M (e). f,g) Changes in absorbance at λ_{abs} = 415 nm (f) and changes in absorbance relative to the absorbance at T = 343 K with fits to the nucleation elongation model (g) plotted against the temperature. Fits were obtained using an individual fitting approach.[4]

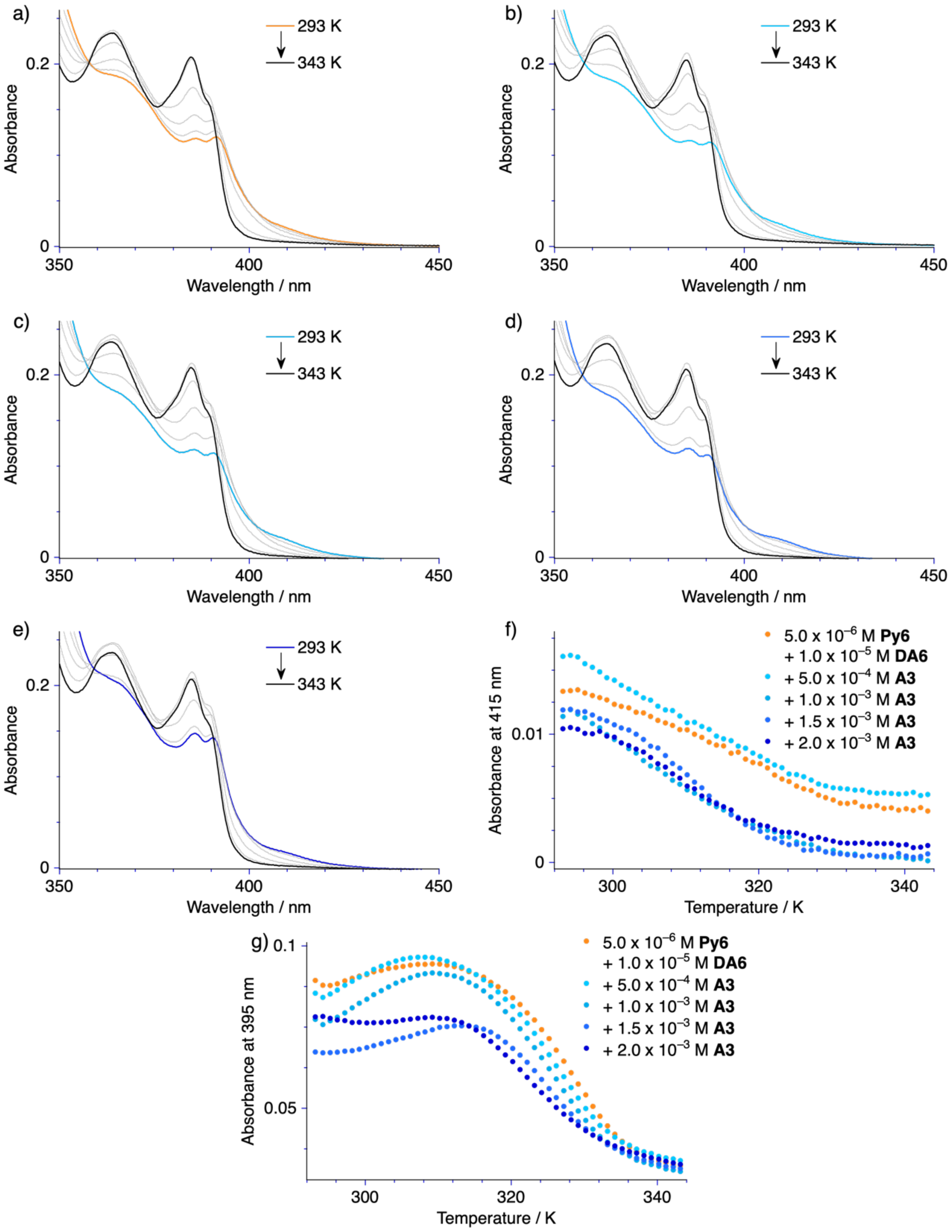

Figure S15. a-e) Temperature-dependent UV-vis absorption spectra of **Py6** (c = 5.0 × 10^{-6} M) and **DA6** (c = 1.0 × 10^{-5} M) in hexane between T = 293 and 333 K using a heating rate of 1 K/min and a data interval of 1 K in a binary mixture (a) as well as in tertiary mixtures with **A3** at c = 5.0 × 10^{-4} M (b); 1.0 × 10^{-3} M (c); 1.5 × 10^{-3} M (d); 2.0 × 10^{-3} M (e). f,g) Changes in absorbance at λ_{abs} = 415 nm (f) and changes in absorbance at λ_{abs} = 395 nm (g) plotted against the temperature.

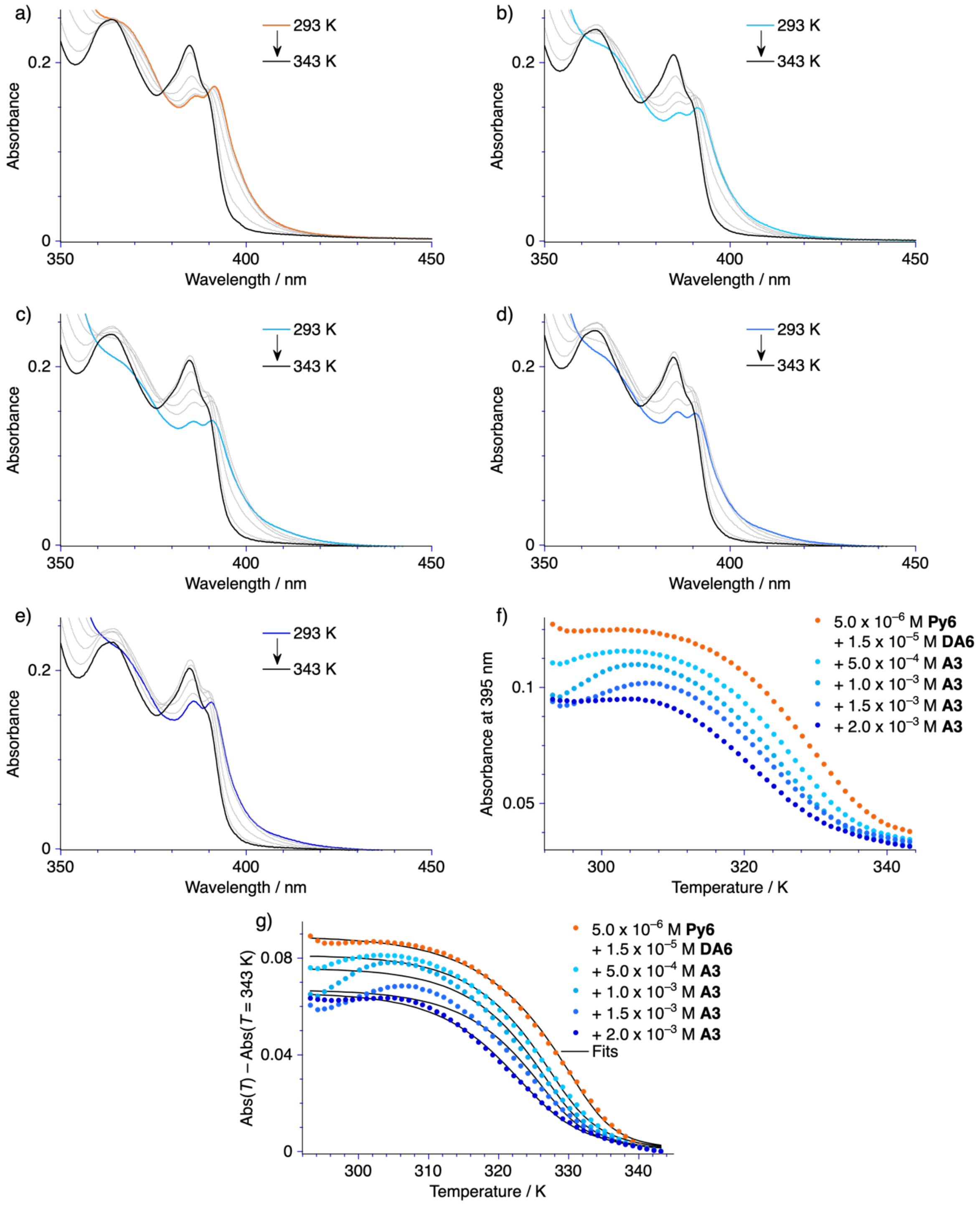

Figure S16. a-e) Temperature-dependent UV-vis absorption spectra of **Py6** (c = 5.0 × 10^{-6} M) and **DA6** (c = 1.5 × 10^{-5} M) in hexane between T = 293 and 333 K using a heating rate of 1 K/min and a data interval of 1 K in a binary mixture (a) as well as in tertiary mixtures with **A3** at c = 5.0 × 10^{-4} M (b); 1.0 × 10^{-3} M (c); 1.5 × 10^{-3} M (d); 2.0 × 10^{-3} M (e). f,g) Changes in absorbance at λ_{abs} = 395 nm (f) and changes in absorbance relative to the absorbance at T = 343 K with fits to the nucleation elongation model (g) plotted against the temperature. Fits were obtained using an individual fitting approach.[4]

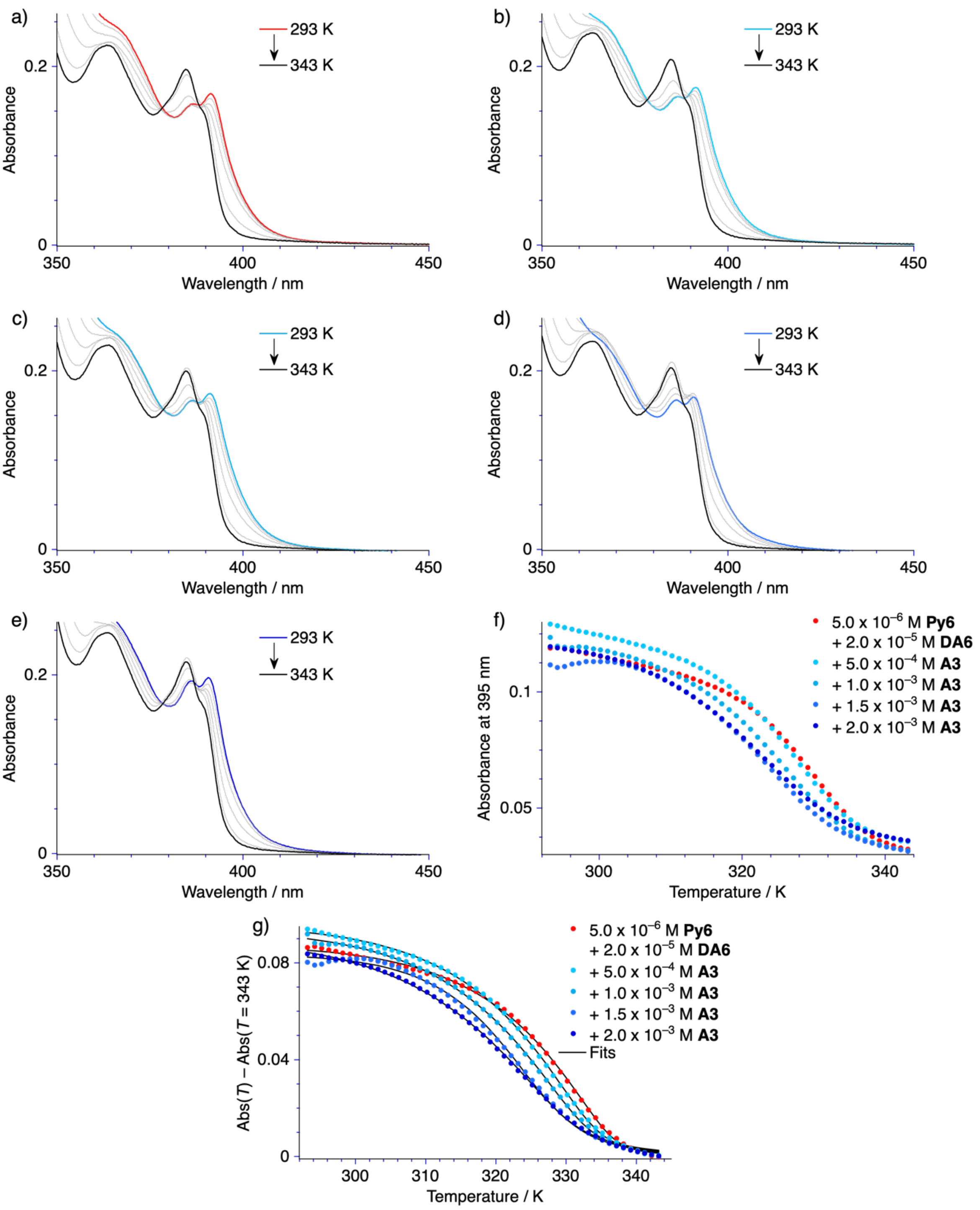

Figure S17. a-e) Temperature-dependent UV-vis absorption spectra of **Py6** (c = 5.0 × 10^{-6} M) and **DA6** (c = 2.0 × 10^{-5} M) in hexane between T = 293 and 333 K using a heating rate of 1 K/min and a data interval of 1 K in a binary mixture (a) as well as in tertiary mixtures with **A3** at c = 5.0 × 10^{-4} M (b); 1.0 × 10^{-3} M (c); 1.5 × 10^{-3} M (d); 2.0 × 10^{-3} M (e). f,g) Changes in absorbance at λ_{abs} = 395 nm (f) and changes in absorbance relative to the absorbance at T = 343 K with fits to the nucleation elongation model (g) plotted against the temperature. Fits were obtained using an individual fitting approach.[4]

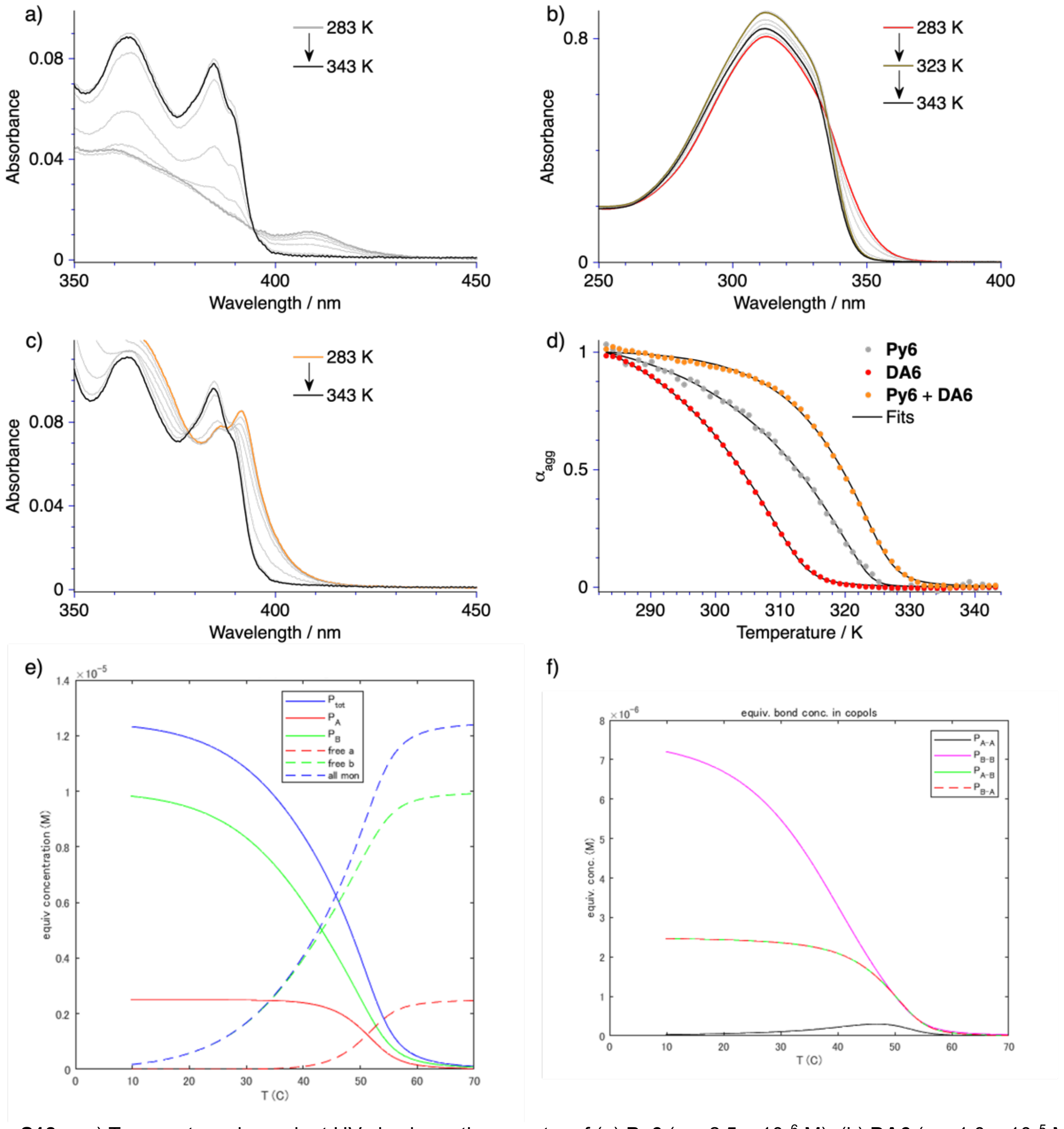

Figure S18. a-c) Temperature-dependent UV-vis absorption spectra of (a) **Py6** (c = 2.5 × 10^{-6} M), (b) **DA6** (c = 1.0 × 10^{-5} M), and (c) a mixture of **Py6** (c = 2.5 × 10^{-6} M) and **DA6** (c = 1.0 × 10^{-5} M) in hexane between T = 283 and 343 K using a heating rate of 1 K/min and a data interval of 1 K. d) Degree of aggregation calculated based on the absorbance at λ_{abs} = 415 nm (**Py6**), λ_{abs} = 345 nm (**DA6**), and λ_{abs} = 395 nm (**Py6** and **DA6**) with fits obtained from the nucleation-elongation model.[4] e,f) Equivalent concentrations (e) and equivalent bond concentrations (f) of the comonomers **Py6** and **DA6** within the copolymer derived from the co-polymerization model.[9]

Table S3. Thermodynamic parameters derived from the copolymerization model shown in Figure S18 (e) and (f).[9]

Binding event	ΔH_e/ kJ mol^{-1}	ΔH_n/ kJ mol^{-1}	ΔS/ kJ mol^{-1} K^{-1}
**Py6-Py6**	–139.0	–9.0	–0.33
**DA6-DA6**	–84.0	–10.0	–0.17
**Py6-DA6**	–130.0	–	–0.30

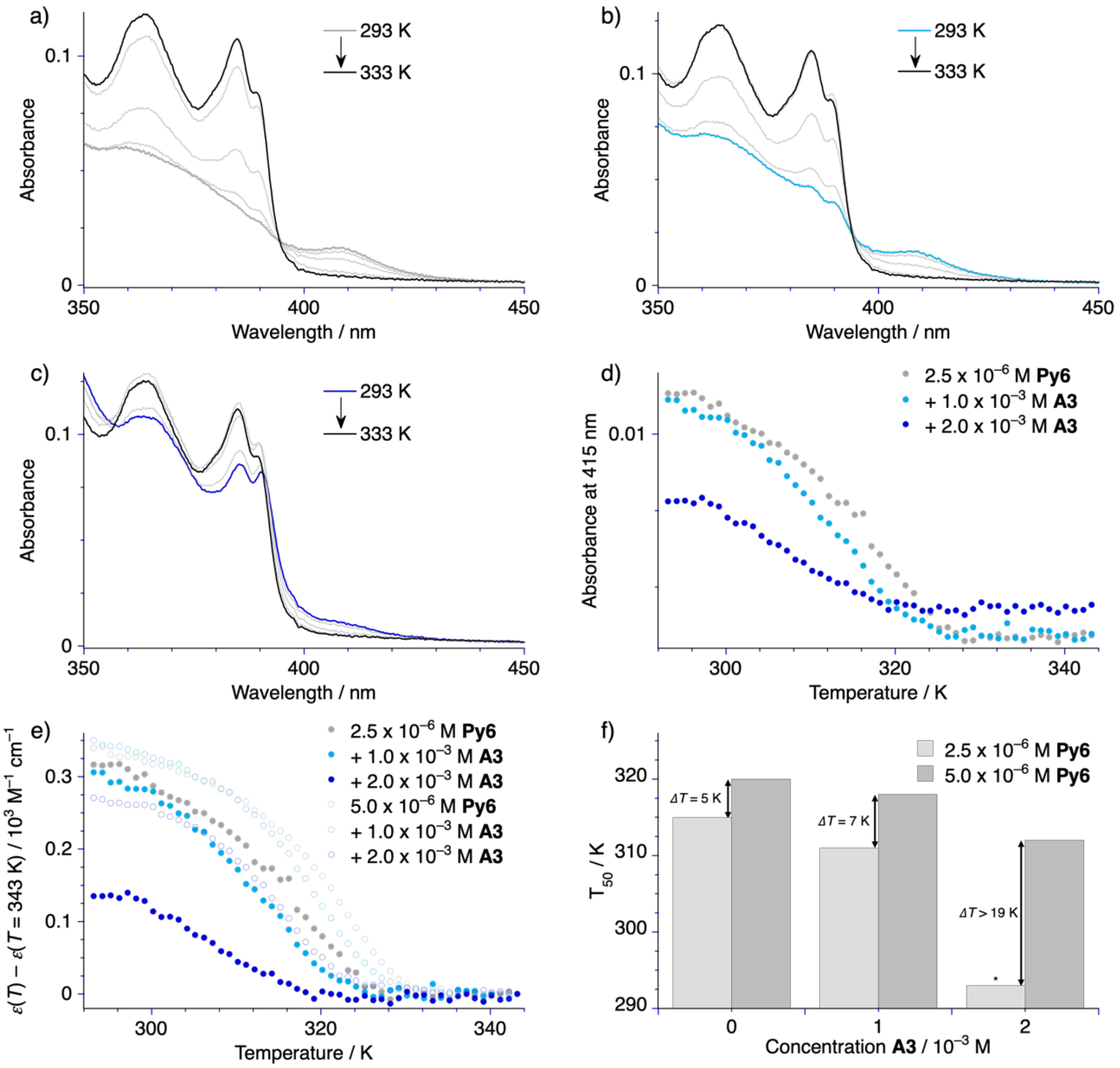

Figure S19. a-c) Temperature-dependent UV-vis absorption spectra of **Py6** ($c = 2.5 \times 10^{-6}$ M) in hexane between $T = 293$ and 333 K using a heating rate of 1 K/min and a data interval of 1 K in isolation (a) as well as in the presence of **A3** at $c = 1.0 \times 10^{-3}$ M (b) and 2.0×10^{-3} M (c). d) Changes in absorbance at $\lambda_{abs} = 415$ nm plotted against the temperature. e) Comparison between the relative temperature-dependent changes in the extinction coefficient determined for $\lambda_{abs} = 415$ nm using low ($c = 2.5 \times 10^{-6}$ M, closed circles) and high ($c = 5.0 \times 10^{-6}$ M, open circles) concentrations of **Py6**. f) Comparison between the T_{50} values of the homopolymer of **Py6** using low ($c = 2.5 \times 10^{-6}$ M, light grey) and high ($c = 5.0 \times 10^{-6}$ M, dark grey) concentrations of **Py6** plotted against the concentration of **A3**. * The relative change in the extinction coefficient at 293 K is already below the threshold for T_{50}, for visual comparison the bar has been plotted for $T_{50} = 293$ K.

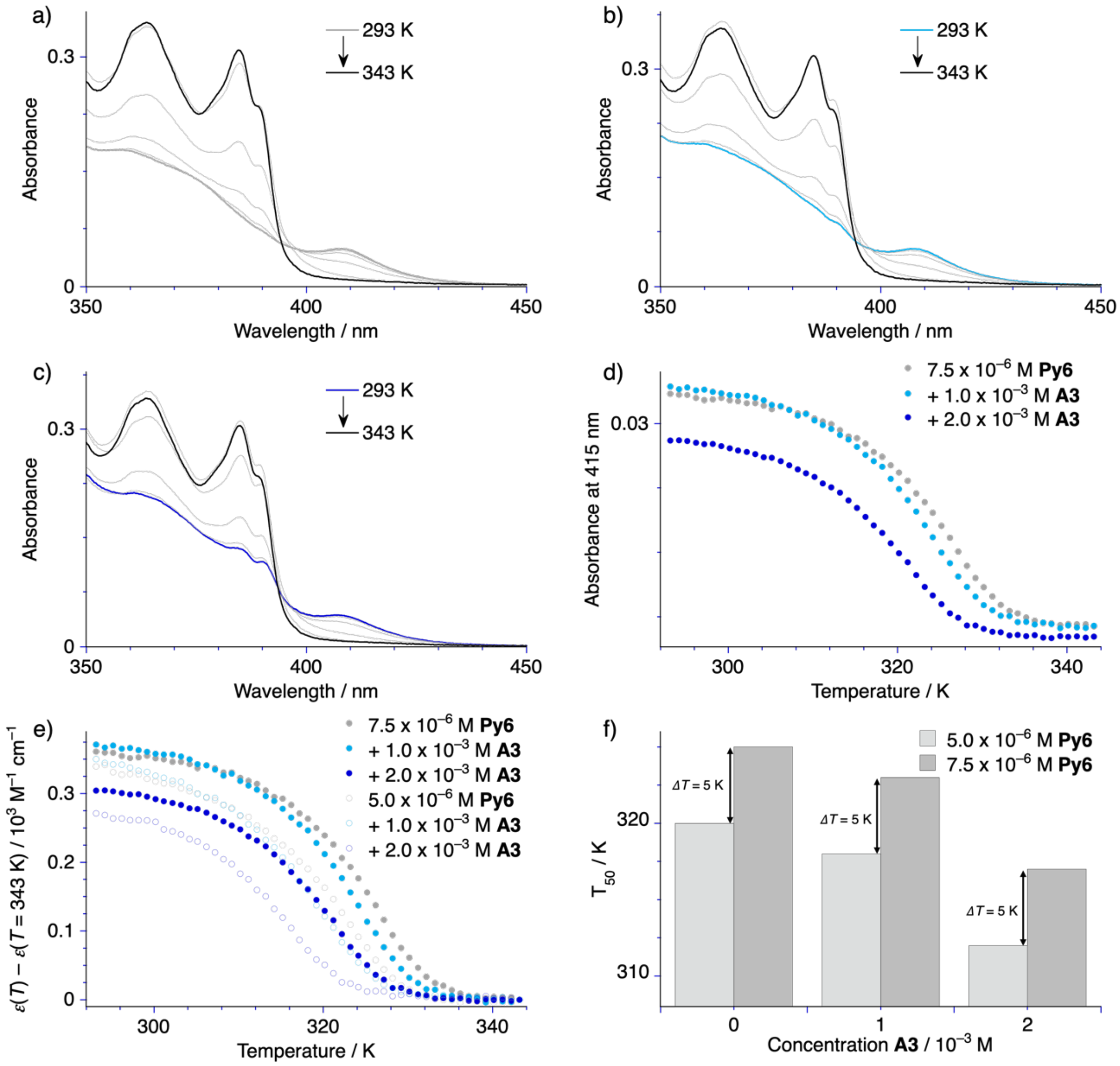

Figure S20. a-c) Temperature-dependent UV-vis absorption spectra of **Py6** (c = 7.5 × 10^{-6} M) in hexane between T = 293 and 333 K using a heating rate of 1 K/min and a data interval of 1 K in isolation (a) as well as in the presence of **A3** at c = 1.0 × 10^{-3} M (b) and 2.0 × 10^{-3} M (c). d) Changes in absorbance at λ_{abs} = 415 nm plotted against the temperature. e) Comparison between the relative temperature-dependent changes in the extinction coefficient determined for λ_{abs} = 415 nm using low (c = 5.0 × 10^{-6} M, open circles) and high (c = 7.5 × 10^{-6} M, closed circles) concentrations of **Py6**. f) Comparison between the T_{50} values of the homopolymer of **Py6** using low (c = 5.0 × 10^{-6} M, light grey) and high (c = 7.5 × 10^{-6} M, dark grey) concentrations of **Py6** plotted against the concentration of **A3**.

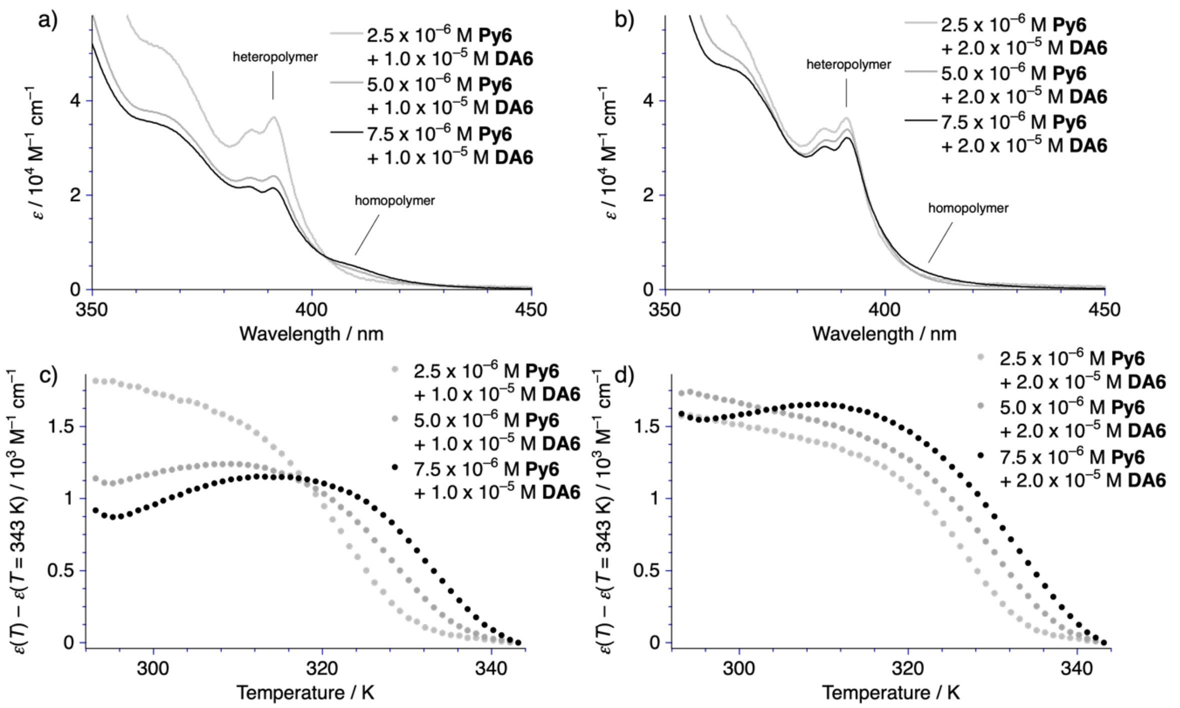

Figure S21. a,b) Comparison between the extinction coefficient of **Py6** at c = 2.5 × 10^{-6} M (light grey), c = 5.0 × 10^{-6} M (dark grey) and c = 7.5 × 10^{-6} M (black) in the presence of **DA6** at c = 1.0 × 10^{-5} M (a) and c = 2.0 × 10^{-5} M (b) in hexane at 293 K. c,d) Comparison between the relative temperature-dependent changes in the extinction coefficient of **Py6** determined for λ_{abs} = 395 nm at c = 2.5 × 10^{-6} M (light grey), c = 5.0 × 10^{-6} M (dark grey) and c = 7.5 × 10^{-6} M (black) in the presence of **DA6** at c = 1.0 × 10^{-5} M (c) and c = 2.0 × 10^{-5} M (d).

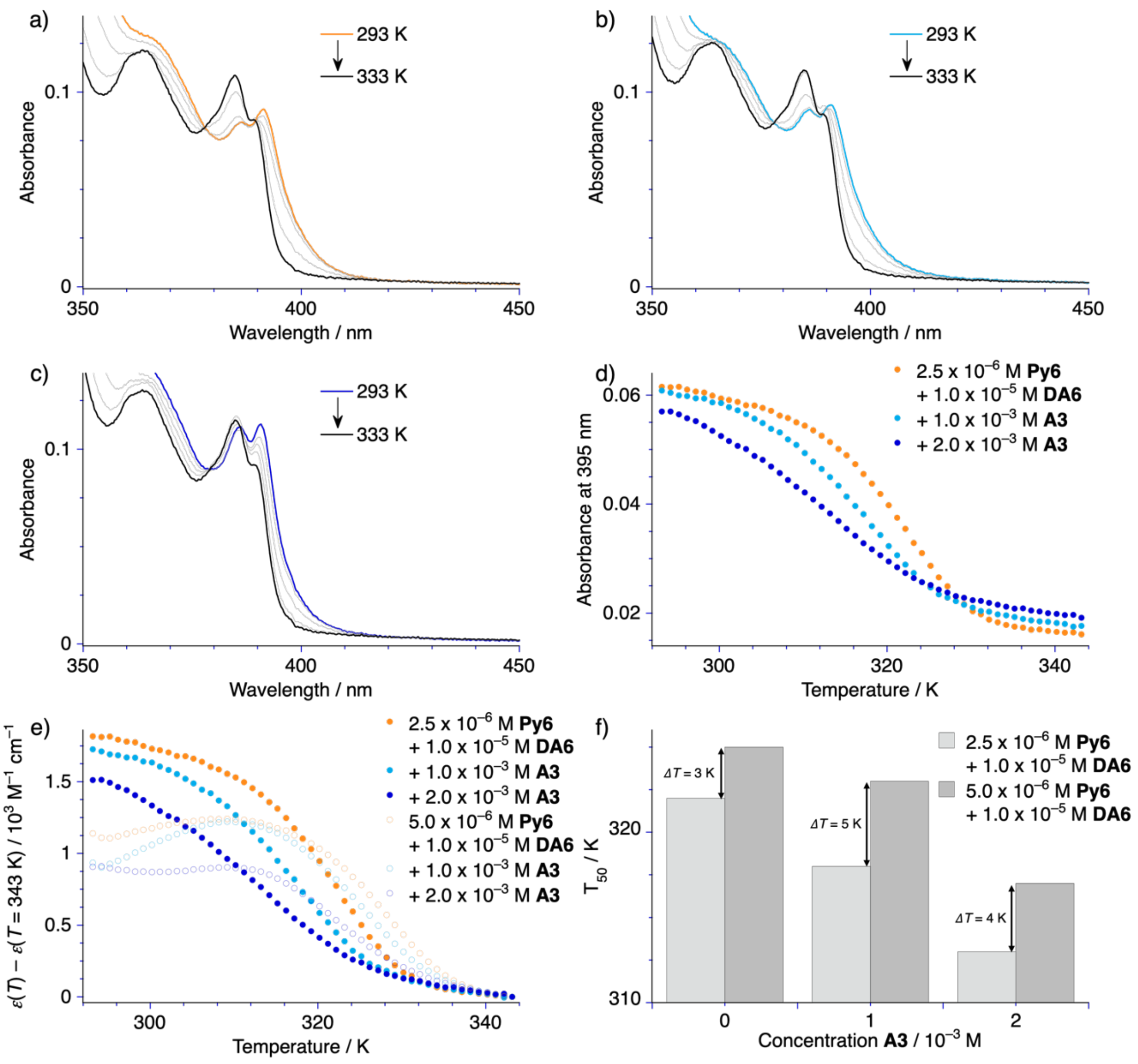

Figure S22. a-c) Temperature-dependent UV-vis absorption spectra of **Py6** ($c = 2.5 \times 10^{-6}$ M) and **DA6** ($c = 1.0 \times 10^{-5}$ M) in hexane between T = 293 and 333 K using a heating rate of 1 K/min and a data interval of 1 K in a binary mixture (a) as well as in tertiary mixtures with **A3** at $c = 1.0 \times 10^{-3}$ M (b) and 2.0×10^{-3} M (c). d) Changes in absorbance at λ_{abs} = 395 nm plotted against the temperature. e) Comparison between the relative temperature-dependent changes in the extinction coefficient determined for λ_{abs} = 395 nm using low ($c = 2.5 \times 10^{-6}$ M, closed circles) and high ($c = 5.0 \times 10^{-6}$ M, open circles) concentrations of **Py6**. f) Comparison between the T_{50} values of the heteropolymer of **Py6** and **DA6** using low ($c = 2.5 \times 10^{-6}$ M, light grey) and high ($c = 5.0 \times 10^{-6}$ M, dark grey) concentrations of **Py6** plotted against the concentration of **A3**.

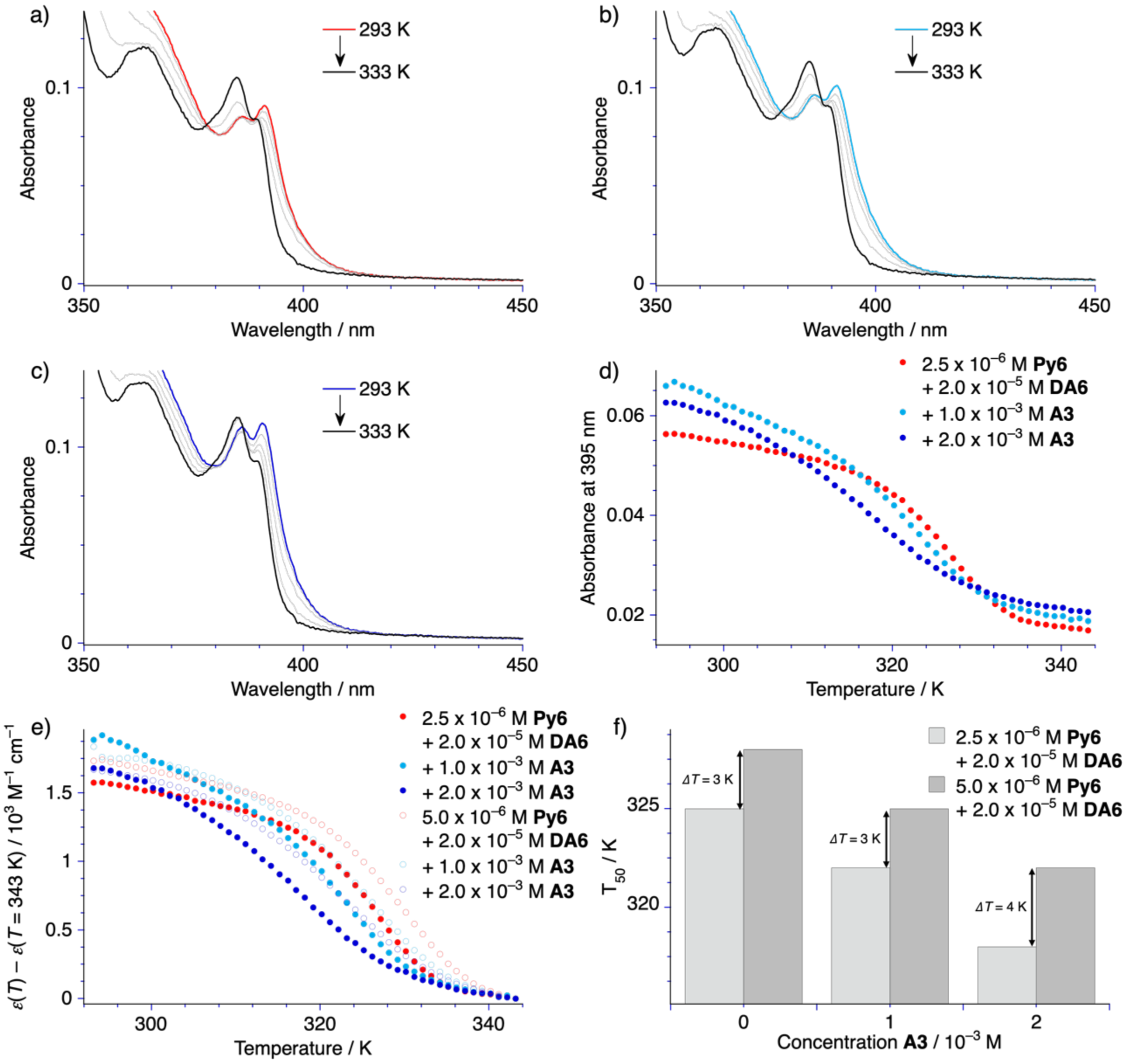

Figure S23. a-c) Temperature-dependent UV-vis absorption spectra of **Py6** (c = 2.5 × 10^{-6} M) and **DA6** (c = 2.0 × 10^{-5} M) in hexane between T = 293 and 333 K using a heating rate of 1 K/min and a data interval of 1 K in a binary mixture (a) as well as in tertiary mixtures with **A3** at c = 1.0 × 10^{-3} M (b) and 2.0 × 10^{-3} M (c). d) Changes in absorbance at λ_{abs} = 395 nm plotted against the temperature. e) Comparison between the relative temperature-dependent changes in the extinction coefficient determined for λ_{abs} = 395 nm using low (c = 2.5 × 10^{-6} M, closed circles) and high (c = 5.0 × 10^{-6} M, open circles) concentrations of **Py6**. f) Comparison between the T_{50} values of the heteropolymer of **Py6** and **DA6** using low (c = 2.5 × 10^{-6} M, light grey) and high (c = 5.0 × 10^{-6} M, dark grey) concentrations of **Py6** plotted against the concentration of **A3**.

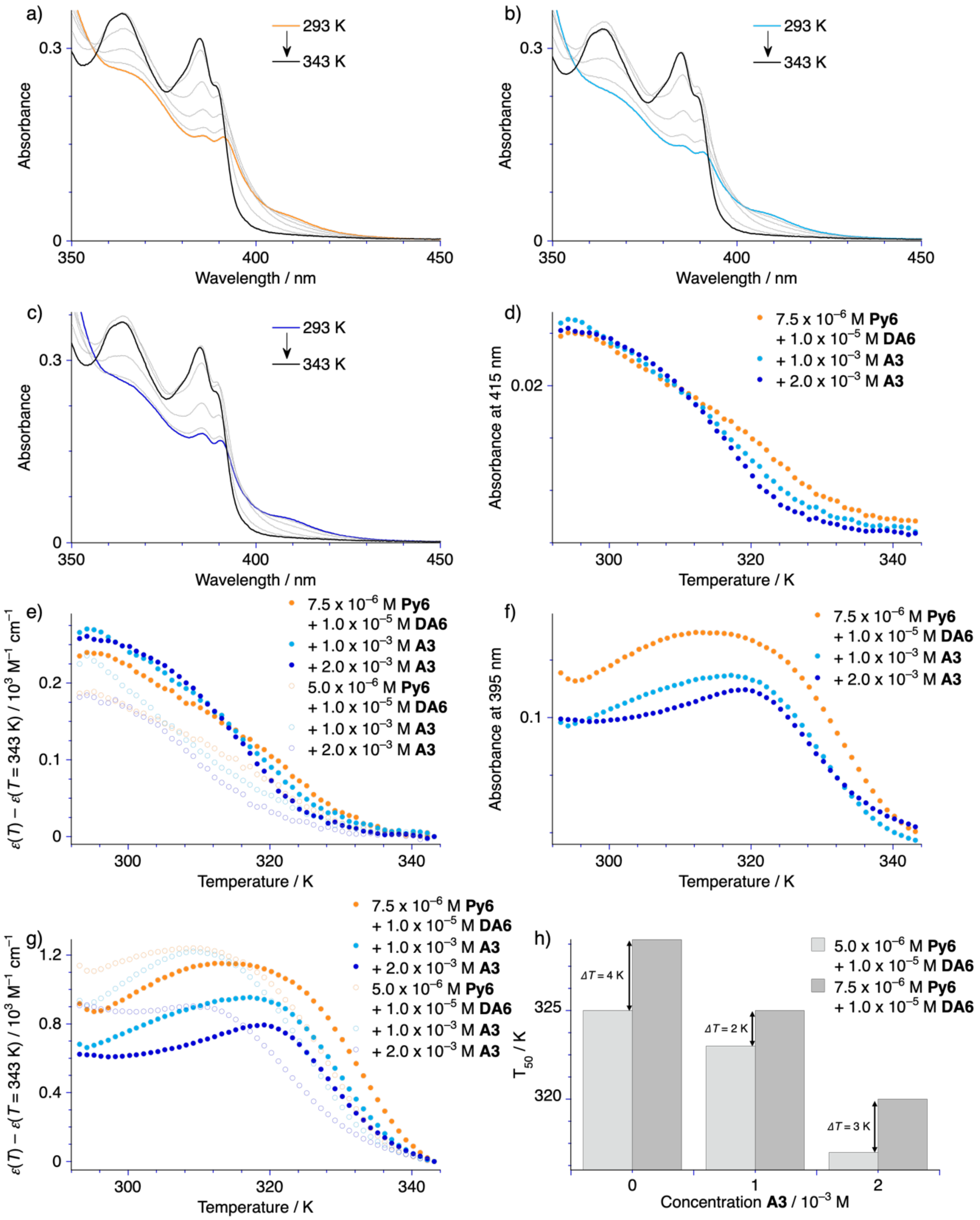

Figure S24. a-c) Temperature-dependent UV-vis absorption spectra of **Py6** (c = 7.5 × 10^{-6} M) and **DA6** (c = 1.0 × 10^{-5} M) in hexane between T = 293 and 333 K using a heating rate of 1 K/min and a data interval of 1 K in a binary mixture (a) as well as in tertiary mixtures with **A3** at c = 1.0 × 10^{-3} M (b) and 2.0 × 10^{-3} M (c). d,f) Changes in absorbance at λ_{abs} = 415 nm (d) and λ_{abs} = 395 nm (f) plotted against the temperature. e,g) Comparison between the relative temperature-dependent changes in the extinction coefficient determined for λ_{abs} = 415 nm (e) and λ_{abs} = 395 nm (g) using low (c = 5.0 × 10^{-6} M, open circles) and high (c = 7.5 × 10^{-6} M, closed circles) concentrations of **Py6**. f) Comparison between the T_{50} values of the heteropolymer of **Py6** and **DA6** using low (c = 5.0 × 10^{-6} M, light grey) and high (c = 7.5 × 10^{-6} M, dark grey) concentrations of **Py6** plotted against the concentration of **A3**.

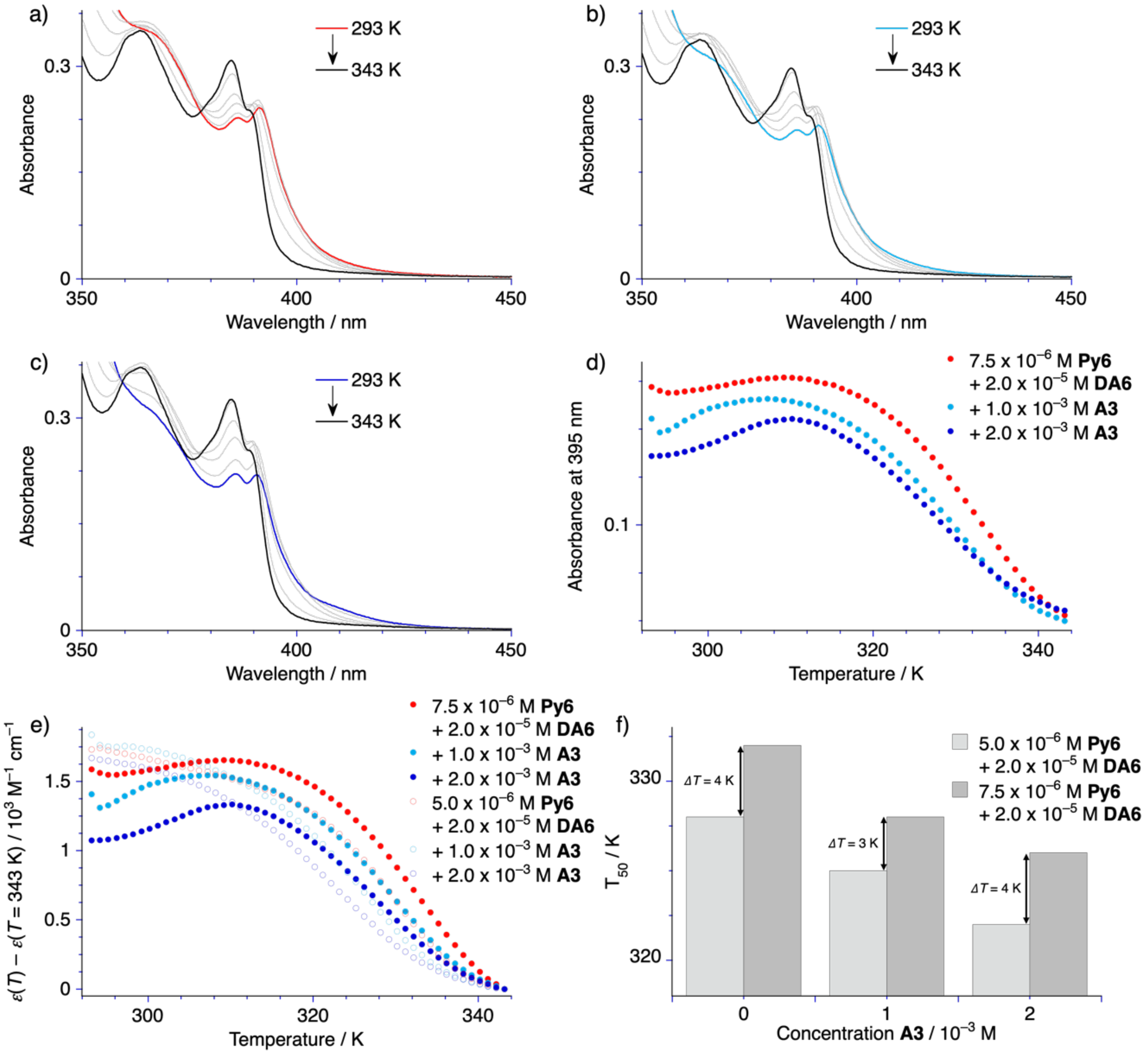

Figure S25. a-c) Temperature-dependent UV-vis absorption spectra of **Py6** ($c = 7.5 \times 10^{-6}$ M) and **DA6** ($c = 2.0 \times 10^{-5}$ M) in hexane between T = 293 and 333 K using a heating rate of 1 K/min and a data interval of 1 K in a binary mixture (a) as well as in tertiary mixtures with **A3** at $c = 1.0 \times 10^{-3}$ M (b) and 2.0×10^{-3} M (c). d) Changes in absorbance at λ_{abs} = 395 nm plotted against the temperature. e) Comparison between the relative temperature-dependent changes in the extinction coefficient determined for λ_{abs} = 395 nm using low ($c = 5.0 \times 10^{-6}$ M, open circles) and high ($c = 7.5 \times 10^{-6}$ M, closed circles) concentrations of **Py6**. f) Comparison between the T_{50} values of the heteropolymer of **Py6** and **DA6** using low ($c = 5.0 \times 10^{-6}$ M, light grey) and high ($c = 7.5 \times 10^{-6}$ M, dark grey) concentrations of **Py6** plotted against the concentration of **A3**.

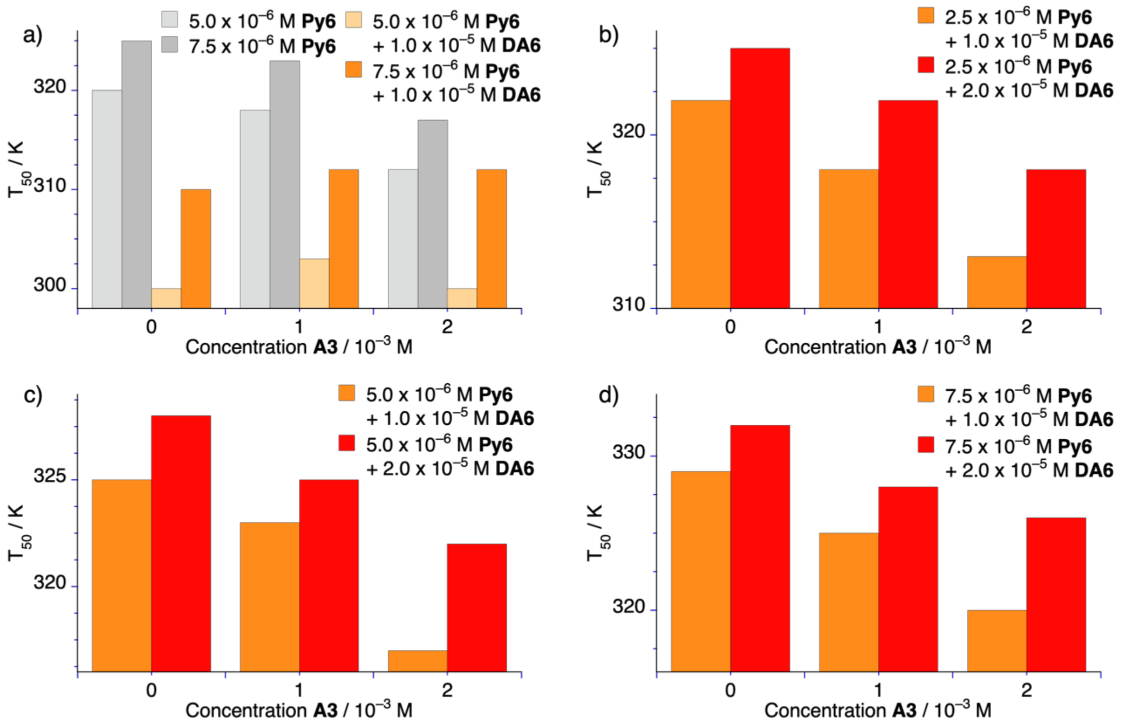

Figure S26. a) Comparison between the T_{50} values of the homopolymer of **Py6** at $c_{\mathbf{Py6}} = 5.0 \times 10^{-6}$ M (light colors) and $c_{\mathbf{Py6}} = 7.5 \times 10^{-6}$ M (dark colors) without **DA6** (grey colors) and with $c_{\mathbf{DA6}} = 1.0 \times 10^{-5}$ M (orange colors) plotted against the concentration of **A3**. b-d) Comparison between the T_{50} values of the heteropolymer of **Py6** and **DA6** at $c_{\mathbf{Py6}} = 2.5 \times 10^{-6}$ M (b), $c_{\mathbf{Py6}} = 5.0 \times 10^{-6}$ M (c) and $c_{\mathbf{Py6}} = 7.5 \times 10^{-6}$ M (d) with $c_{\mathbf{DA6}} = 1.0 \times 10^{-5}$ M (orange) and $c_{\mathbf{DA6}} = 2.0 \times 10^{-5}$ M (red) plotted against the concentration of **A3**.

Supplementary discussion 2

As **DA6** has the same aggregation inducing moiety in its molecular design as **Py6**, **A3** can act as a sequestrator by identical mechanistic pathways. (Figure S27). By analyzing the secondary plots of the disassembly process upon heating (Figure S27f), a decrease in the elongation temperature can be observed. In comparison to **Py6** however, the influence of **A3** on **DA6** is significant enough to make the fitting procedure produce unsatisfactory results when the additive concentration exceeds 1.0 × 10^{-3} M and the corresponding fits should not be analyzed quantitatively. This behavior can be rationalized by again analyzing the underlying mass balance equations (Figure S28, equation 1 and 2).

As the equilibrium constant regarding the sequestration (K^S) are likely comparable to that of the binary mixture of **Py6** and **A3**, the enhanced influence of the sequestrator should be attributed to the lower binding affinity of **DA6** compared to **Py6** in the supramolecular homo-polymerization (Table S1), leading to a shift of the concerted equilibria towards the sequestered species.

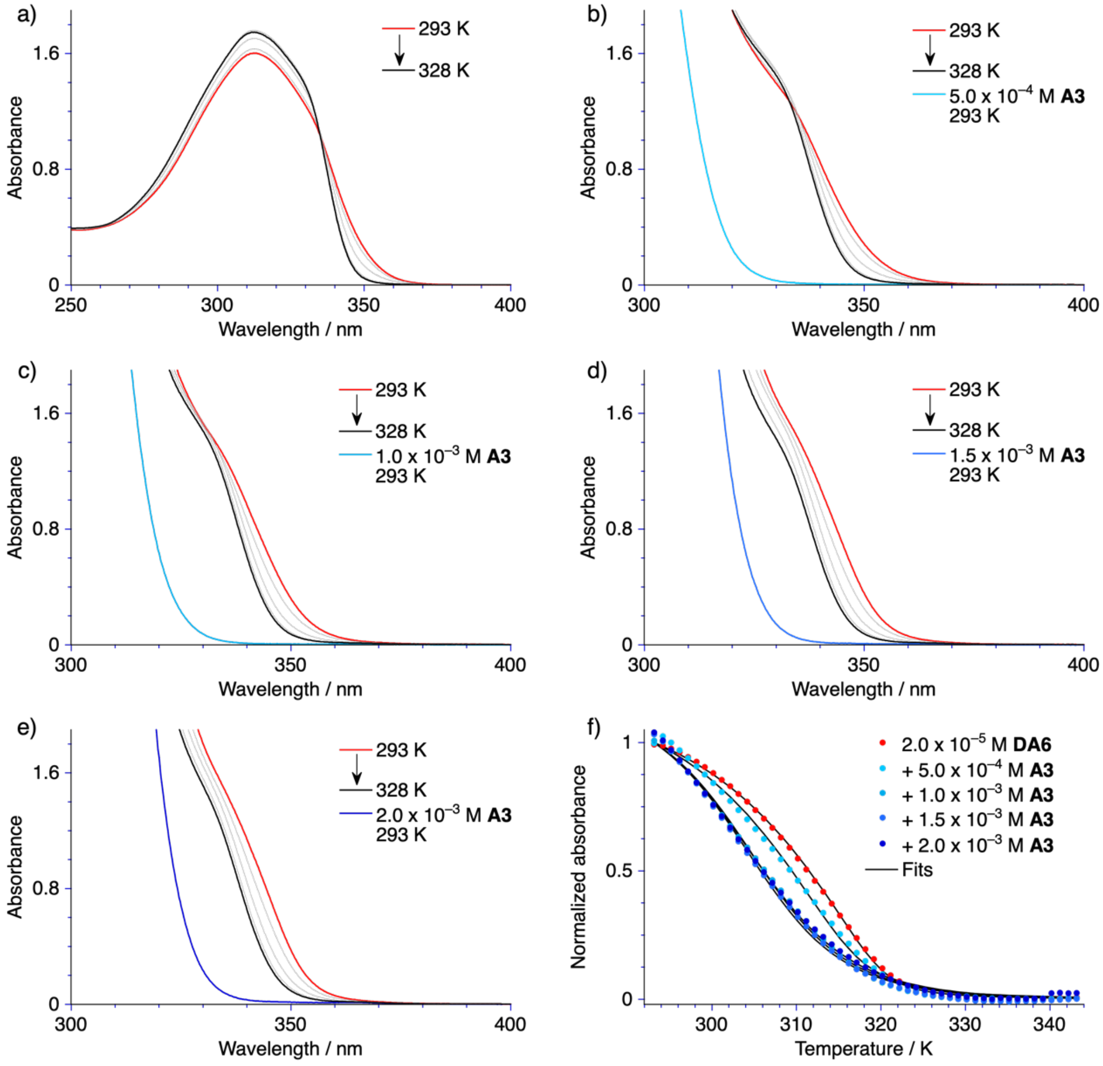

Figure S27. a-e) Temperature-dependent UV-vis absorption spectra of **DA6** (c = 2.0 × 10^{-5} M) in hexane between T = 293 K and 328 K using a heating rate of 1 K/min (a) as well as in binary with **A3** at c = 5.0 × 10^{-4} M (b), 1.0 × 10^{-3} M (c), 1.5 × 10^{-3} M (d), and 2.0 × 10^{-3} M (e). The spectra of **A3** at the corresponding concentrations in hexane at T = 293 K have been shown to demonstrate the minor degree of spectral overlap at λ_{abs} = 350 nm. f) Normalized changes in absorbance at λ_{abs} = 350 nm plotted against the temperature with fits obtained from the nucleation elongation model.[4]

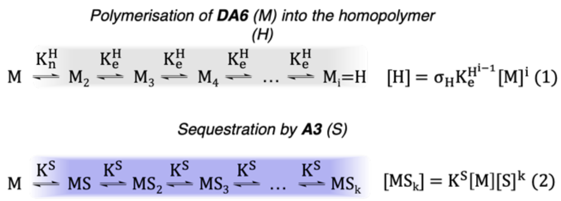

Figure S28. Equilibria describing the homo-polymerization of **DA6** (M) into the homopolymer H (Equation 1) and the sequestration by **A3** (S in Equation 2). σ_H corresponds to the cooperativity factor of the homo-polymerization, the subscript *i* corresponds to the number of monomers M in a homopolymer or copolymer and the subscript *k* corresponds to the number of sequestrators S bound to a single molecule of the monomer M.

References

[S1] S. S. Janni, M. K. Manheri, *Langmuir* **2013**, *29*, 15182–15190.
[S2] K. Hirai, K. Hatanaka, T. Yamaguchi, A. Miyajima, T. Kitagawa, H. Tomioka, *J. Phys. Org. Chem.* **2011**, *24*, 909–920.
[S3] A. Y.-Y. Tam, K. M.-C. Wong, N. Zhu, G. Wang, V. W.-W. Yam, *Langmuir* **2009**, *25*, 8685–8695.
[S4] H. M. M. ten Eikelder, A. J. Markvoort, T. F. A. de Greef, P. A. J. Hilbers, *J. Phys. Chem. B* **2012**, *116*, 5291–5301.
[S5] F. Helmich, C. C. Lee, M. M. L. Nieuwenhuizen, J. C. Gielen, P. C. M. Christianen, A. Larsen, G. Fytas, P. E. L. G. Leclère, A. P. H. J. Schenning, E. W. Meijer, *Angew. Chem. Int. Ed.* **2010**, *49*, 3939–3942.
[S6] E. Weyandt, L. Leanza, R. Capelli, G. M. Pavan, G. Vantomme, E. W. Meijer, *Nat. Commun.* **2022**, *13*, 248.
[S7] S. A. H. Jansen, E. Weyandt, T. Aoki, T. Akiyama, Y. Itoh, G. Vantomme, T. Aida, E. W. Meijer, *J. Am. Chem. Soc.* **2023**, *145*, 4231–4237.
[S8] B. Adelizzi, I. A. W. Filot, A. R. A. Palmans, E. W. Meijer, *Chem. Eur. J.* **2016**, *23*, 6103-6110.
[S9] H. M. M. ten Eikelder, B. Adelizzi, A. R. A. Palmans, A. J. Markvoort, *J. Phys. Chem. B* **2019**, *123*, 6627-6642.

NMR spectroscopy

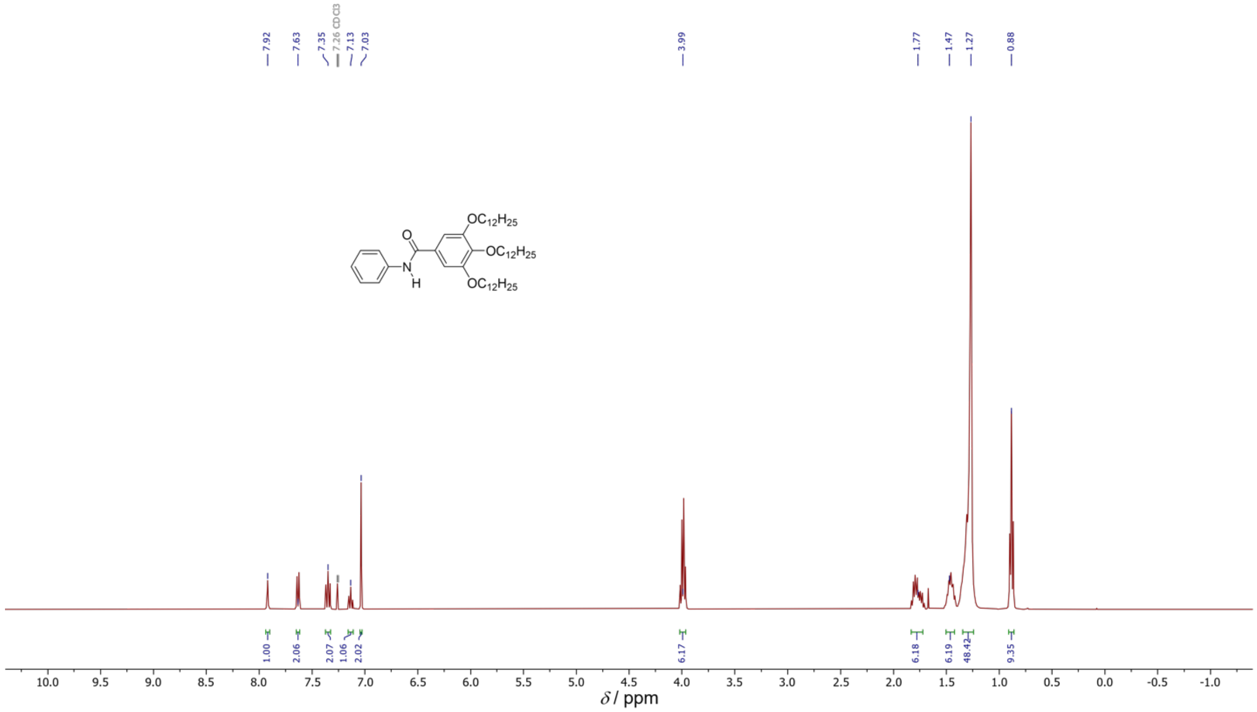

Figure S29. ^{1}H NMR spectrum of compound **A3** (400 MHz, 298 K, $CDCl_3$).

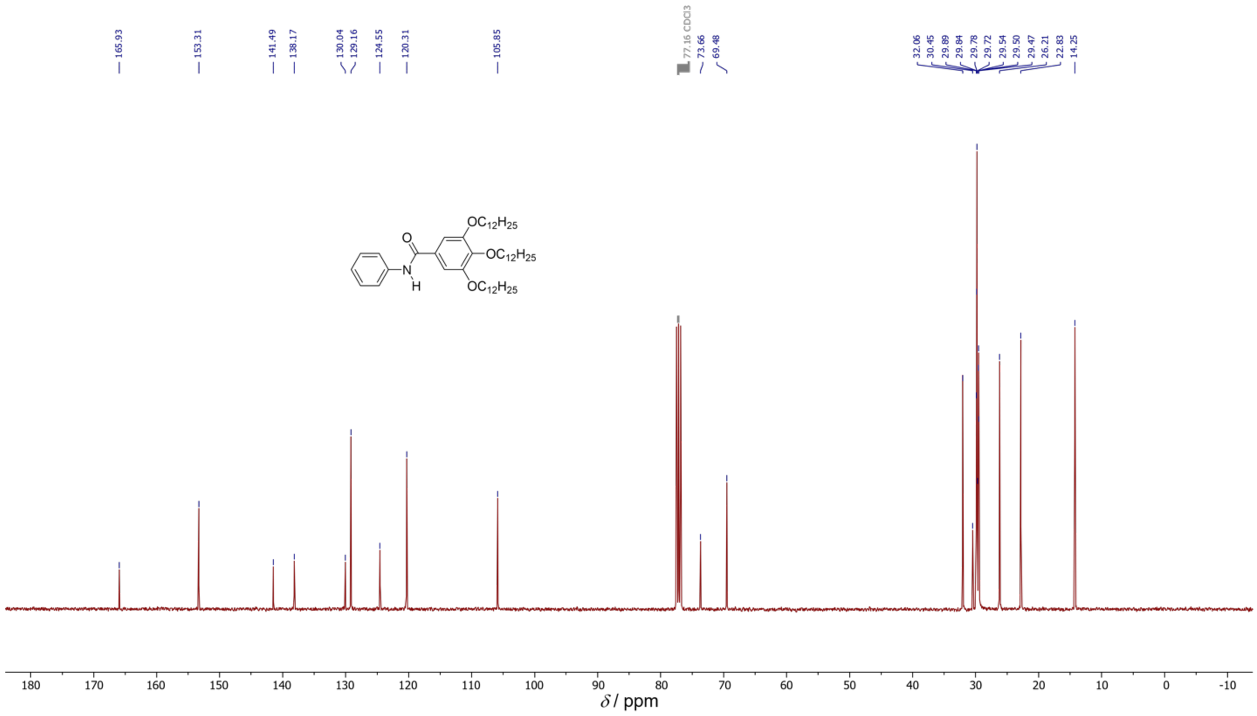

Figure S30. ^{13}C NMR spectrum of compound **A3** (100 MHz, 298 K, $CDCl_3$).

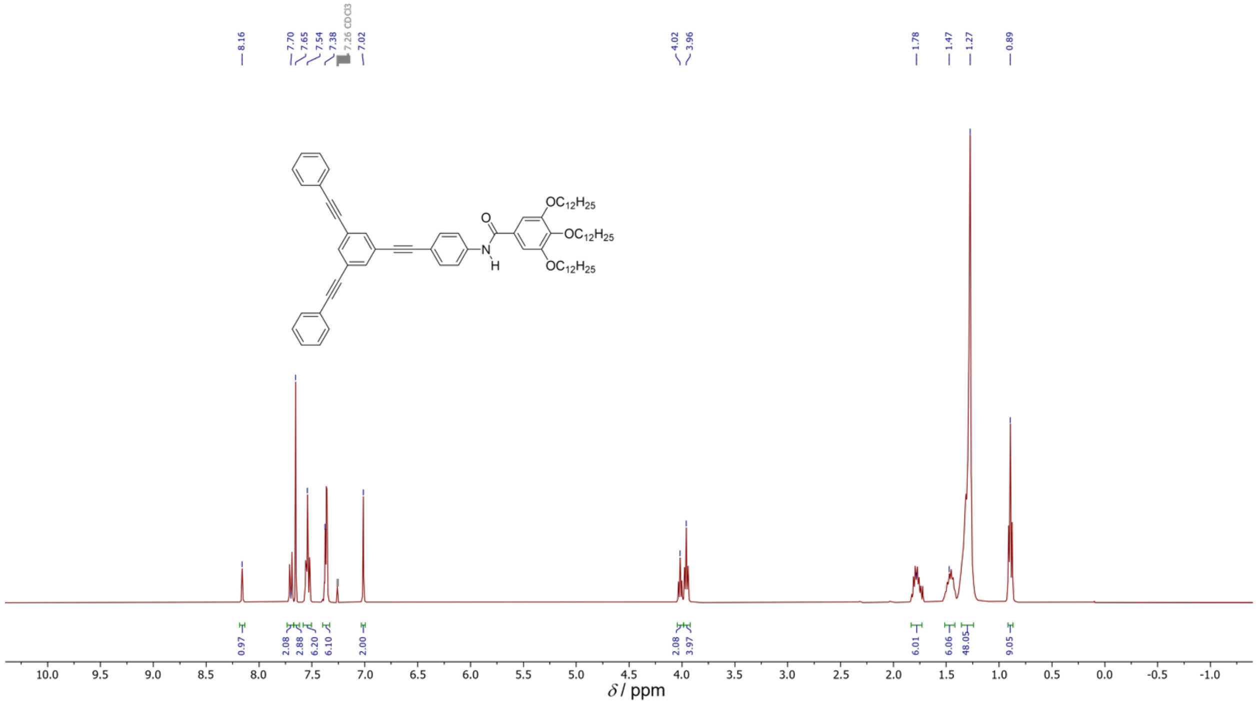

Figure S31. ^{1}H NMR spectrum of compound **TB3** (400 MHz, 298 K, $CDCl_3$).

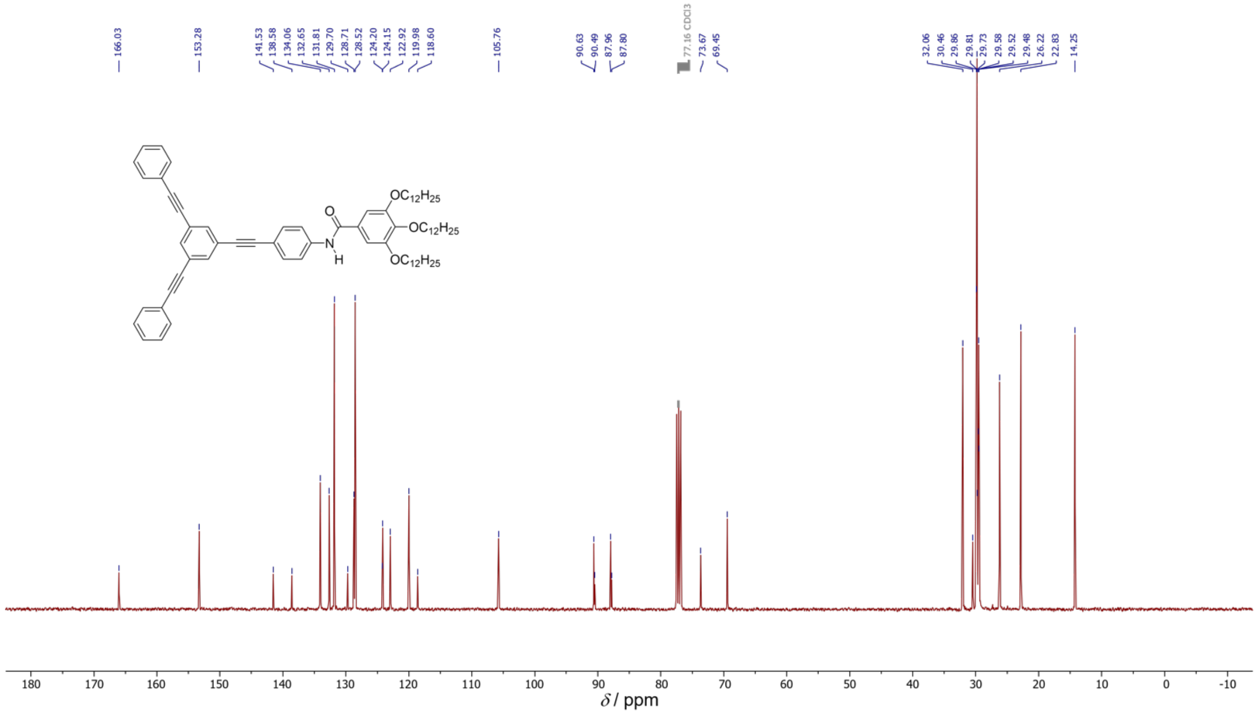

Figure S32. ^{13}C NMR spectrum of compound **TB3** (100 MHz, 298 K, $CDCl_3$).

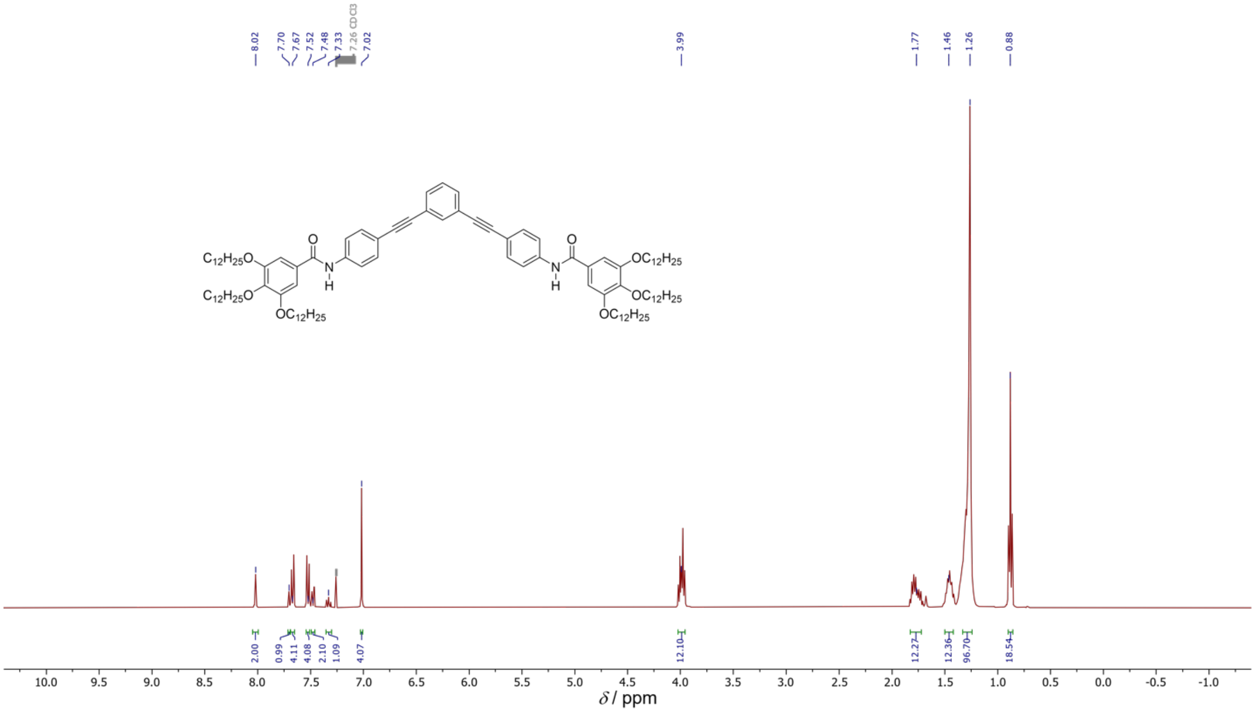

Figure S33. ^{1}H NMR spectrum of compound **DA6** (400 MHz, 298 K, $CDCl_3$).

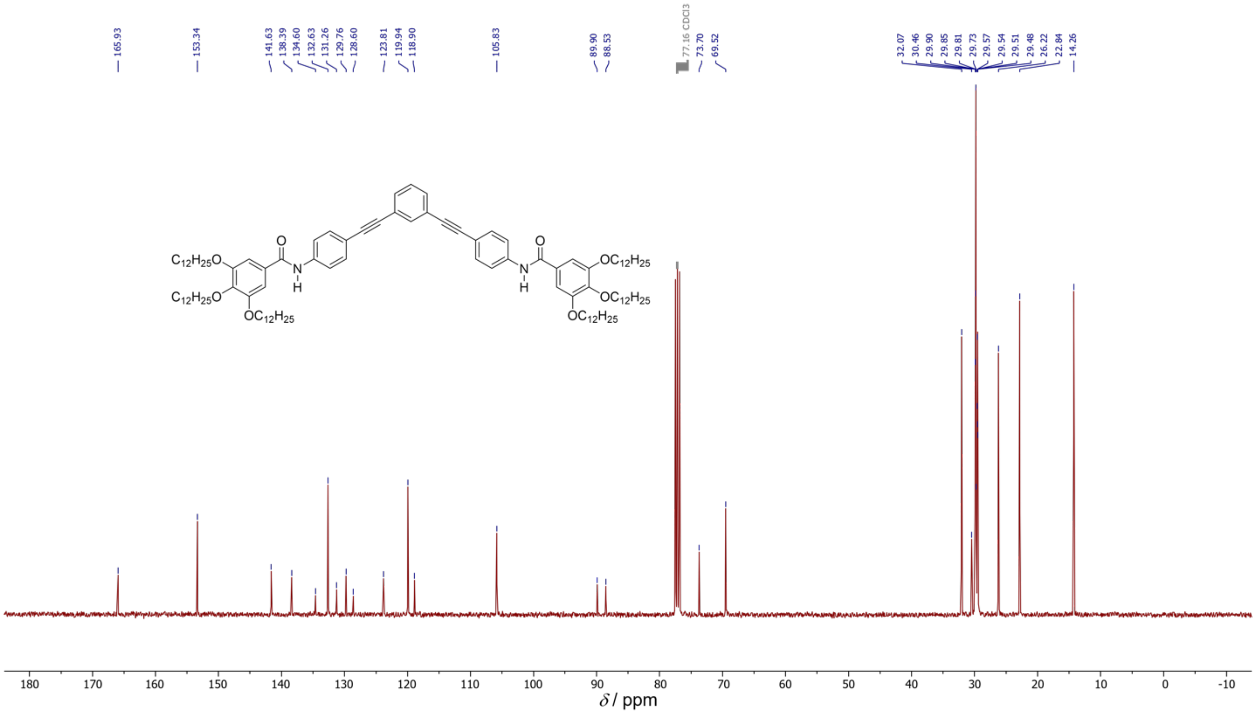

Figure S34. ^{13}C NMR spectrum of compound **DA6** (100 MHz, 298 K, $CDCl_3$).
